# Lewis Acid‐Catalyzed Diels‐Alder Reactions: Reactivity Trends across the Periodic Table

**DOI:** 10.1002/chem.202100522

**Published:** 2021-05-01

**Authors:** Pascal Vermeeren, Marco Dalla Tiezza, Michelle van Dongen, Israel Fernández, F. Matthias Bickelhaupt, Trevor A. Hamlin

**Affiliations:** ^1^ Department of Theoretical Chemistry Amsterdam Institute of Molecular and Life Sciences (AIMMS) Amsterdam Center for Multiscale Modeling (ACMM) Vrije Universiteit Amsterdam De Boelelaan 1083 1081 HV Amsterdam (The Netherlands; ^2^ Departamento de Química Orgánica I and Centro de Innovación en Química Avanzada (ORFEO-CINQA) Facultad de Ciencias Químicas Universidad Complutense de Madrid 28040 Madrid Spain; ^3^ Institute for Molecules and Materials Radboud University Heyendaalseweg 135 6525 AJ Nijmegen (The Netherlands

**Keywords:** activation strain model, density functional calculations, Diels-Alder reaction, Lewis acids, reactivity

## Abstract

The catalytic effect of various weakly interacting Lewis acids (LAs) across the periodic table, based on hydrogen (Group 1), pnictogen (Group 15), chalcogen (Group 16), and halogen (Group 17) bonds, on the Diels‐Alder cycloaddition reaction between 1,3‐butadiene and methyl acrylate was studied quantum chemically by using relativistic density functional theory. Weakly interacting LAs accelerate the Diels‐Alder reaction by lowering the reaction barrier up to 3 kcal mol^−1^ compared to the uncatalyzed reaction. The reaction barriers systematically increase from halogen<hydrogen<chalcogen<pnictogen‐bonded LAs, *i. e*., the latter have the least catalytic effect. Our detailed activation strain and Kohn‐Sham molecular orbital analyses reveal that these LAs lower the Diels‐Alder reaction barrier by increasing the asynchronicity of the reaction to relieve the otherwise destabilizing Pauli repulsion between the closed‐shell filled π‐orbitals of diene and dienophile. Notably, the reactivity can be further enhanced on going from a Period 3 to a Period 5 LA, as these species amplify the asynchronicity of the Diels‐Alder reaction due to a stronger binding to the dienophile. These findings again demonstrate the generality of the *Pauli repulsion‐lowering catalysis* concept.

## Graphical abstract

**Periodic reactivity trends**! Quantum chemical activation strain analyses reveal how various weakly interacting Lewis acids across the periodic table, based on hydrogen, pnictogen, chalcogen, and halogen bonds, accelerate the Diels‐Alder reaction between 1,3‐butadiene and methyl acrylate by amplifying the degree of asynchronicity and reducing the Pauli repulsion between the reactants.

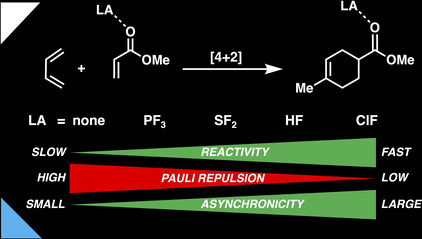

## Introduction

The Diels‐Alder (DA) cycloaddition reaction is of paramount importance in synthetic organic chemistry.^[1]^ Since its discovery in 1928,^[2]^ it has paved the way for a convenient procedure to create six‐membered rings, with up to four stereocenters, becoming the gold standard for many applications ranging from the synthesis of natural products to the industrial production of relevant compounds in the pharmaceutical field.^[3]^ A striking number of organocatalysts have been developed over the years to increase the reactivity and selectivity of the DA reaction. For example, Lewis acids (LAs) are able to greatly accelerate the DA via binding to the dienophile.^[4]^ Interestingly, LAs can also reverse the regiochemical course of the DA reaction leading to products that otherwise would be impossible to synthesize:^[5]^ a pioneering example has been shown by Kishi et al. on the total synthesis of the tetrodotoxin.^[6]^

**Scheme 1:**
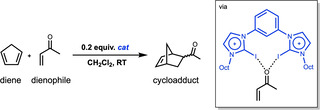
The Diels‐Alder reaction between cyclopentadiene (diene) and 2‐butenone (dienophile) catalyzed by a bifunctional halogen‐bonded catalyst, as shown by Jungbauer et al.^[12e]^

In analogy with conventional Lewis acids, hydrogen‐bonded organocatalysts are also able to significantly modify both the reactivity and selectivity of the DA reaction. For example, hydrogen‐bonded solvents can activate ketones to undergo a DA reaction,^[7]^ or bifunctional hydrogen bond donor organocatalysts, such as differently substituted thioureas, are able to increase both the reaction rate as well as the *endo*/*exo*‐selectivity of DA reactions between a diene and α, β‐unsaturated carbonyl compounds.[^8^, ^9a^] Similar catalytic effects were also reported using catalysts featuring similar weak interactions such as pnictogen,^[10]^ chalcogen,^[11]^ and halogen bonds (Scheme 1).[^12^, ^13^] Moreover, in our previous work, we have shown that Lewis acidic alkali cations can also exert a remarkable catalytic effect on the generally slow aromatic DA reaction: the archetypal aromatic cycloaddition reaction between benzene and acetylene can be effectively accelerated by up to 5 orders of magnitude.^[9b]^ Thus, in general, weakly interacting Lewis acids play a crucial role in the reactivity of organic reactions, and hence it is highly important to understand the driving force behind this class of catalysis.

The catalytic ability of Lewis acids, such as hydrogen, halogen, chalcogen, and pnictogen bonded species, is traditionally attributed to the lowering of the LUMO of the activated dienophile, resulting in a smaller, *i. e*., more favorable, HOMO_diene_‐LUMO_dienophile_ energy gap and hence stronger orbital interaction.^[14]^ Recently, however, we have shown that this rationale is incomplete. Our analyses revealed that, although the LUMO of the dienophile becomes indeed stabilized upon binding to the LA, the increase in reaction rate is determined by a significant reduction of Pauli repulsion between the occupied π‐molecular orbitals of the two reactants.^[9]^ Binding of a LA to the dienophile not only polarizes the occupied π‐orbital density on the C=C double bond of the dienophile away from the incoming diene, but it also introduces an asymmetry in this orbital, amplifying the asynchronicity of the DA reaction. Both effects contribute to diminishing the Pauli repulsion, however, the latter effect also results in less pressure on the reactants to deform and hence a less destabilizing activation strain in the transition state. We argue that the concept of *Pauli repulsion‐lowering catalysis* is also operative in LA‐catalyzed Diels‐Alder reactions where the Lewis acid is weakly bonded to the dienophile via hydrogen, halogen, chalcogen, or pnictogen bonds.

**Scheme 2:**
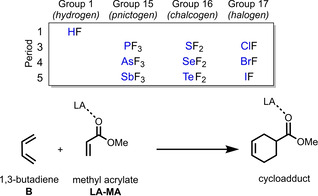
The uncatalyzed (LA=none) and Lewis acid‐catalyzed Diels‐Alder reactions between 1,3‐butadiene (**B**) and (LA‐)methyl acrylate (**LA‐MA**).

To this end, we have investigated the periodic reactivity trends of the hydrogen (Group 1), pnictogen (Group 15), chalcogen (Group 16), and halogen (Group 17) bond mediated Diels‐Alder (DA) cycloaddition reaction between 1,3‐butadiene (**B**) and methyl acrylate (**MA**) (Scheme 2), exclusively following the more favorable *endo* reaction pathway,^[15]^ by means of the activation strain model and quantitative Kohn‐Sham molecular orbital theory. This computational methodology has been proven to be reliable for the understanding of fundamental processes in organic chemistry.[^9^, ^15^] First, we quantitatively examine the bonding between the Lewis acid **AF_n_** (A=H with n=1, A=Cl/Br/I with n=1, A=S/Se/Te with n=2, A=P/As/Sb with n=3) and **MA** leading to the activated dienophile (**LA‐MA**). Secondly, we analyze the catalytic effect of the Lewis acidic hydrogen, halogen, chalcogen, and pnictogen bonds on the DA reactivity of **B** and the **LA‐MA** complex. At last, we study how the reactivity changes upon going from Period 3 to Period 5 Lewis acids.

## Computational Methods

### Computational details

All calculations were performed using the Amsterdam Density Functional (ADF2018.105) software package.^[16]^ The generalized gradient approximation (GGA) functional BP86 was used for the optimizations of all stationary points as well as the analyses.^[17]^ The basis set employed, denoted TZ2P,^[18]^ is of triple‐ζ quality and is augmented with two sets of polarization functions on each atom. Scalar relativistic effects are accounted for using the zeroth‐order regular approximation (ZORA).^[19]^ This level of theory has been proven to be accurate in calculating the relative trends in reaction barriers and energies as well as performing the activation strain and energy decomposition analyses for cycloaddition reactions.[^9b^, ^20^] The accuracies of the fit scheme (Zlm fit)^[21a]^ and the integration grid (Becke grid)^[21b]^ were set to VERYGOOD. In addition, geometries and energies were recomputed using ZORA‐BP86‐D3(BJ)/TZ2P^[22]^ to access the effect of dispersion on the reactivity trends. Equilibrium and transition state geometries were verified by means of vibrational analysis, to assess the nature of all structures: for minima, no imaginary frequencies were found, whereas transition states present a single imaginary frequency. Besides, the character of the normal mode associated with the imaginary frequency was analyzed to ensure that the correct transition state was found, *i. e*., the formation of the two C−C bonds between the reactants. The potential energy surfaces of the studied Diels‐Alder reactions were obtained by utilizing intrinsic reaction coordinate (IRC) calculations.^[23]^ The acquired potential energy surfaces were analyzed by using the PyFrag 2019 program.^[24]^ Optimized structures were illustrated using CYLview.^[25]^

### Activation Strain Model and Energy Decomposition Analysis

The activation strain model of chemical reactivity (ASM,^[26]^ also known as the distortion/interaction model^[27]^), is a fragment‐based approach based on the idea that the energy of a reacting system, *i. e*., the potential energy surface, is described with respect to, and understood in terms of the characteristics of, the original reactants. It considers their rigidity and the extent to which the reactants must deform during the reaction plus their capability to interact as the reaction proceeds. In this model, we decompose the total energy, Δ*E*(ζ), into the respective total strain and interaction energy, Δ*E*_strain_(ζ) and Δ*E*_int_(ζ), and project these values onto the reaction coordinate ζ [Eq. 1].

(1) $$\Delta E\left(\zeta \right)=\Delta E{}_{\mathrm{strain}}\left(\zeta \right)+\Delta E{}_{\mathrm{int}}\left(\zeta \right)$$

In this equation, the total strain energy, Δ*E*_strain_(ζ), is the penalty that needs to be paid to deform the reactants from their equilibrium structure to the geometry they adopt during the reaction at point ζ of the reaction coordinate. On the other hand, the interaction energy, Δ*E*_int_(ζ), accounts for all the chemical interactions that occur between the deformed fragments along the reaction coordinate.

The interaction energy between the deformed reactants is further analyzed in terms of our canonical energy decomposition analysis (EDA).^[28]^ The EDA decomposes the ▵*E*_int_(ζ) into the following three physically meaningful energy terms [Eq. 2]:

(2) $$\Delta E{}_{\mathrm{int}}\left(\zeta \right)\;=\;\Delta V{}_{\mathrm{elstat}}\left(\zeta \right)+\Delta E{}_{\mathrm{Pauli}}\left(\zeta \right)+\Delta E{}_{\mathrm{oi}}\left(\zeta \right)$$

Herein, Δ*V*_elstat_(ζ) is the classical electrostatic interaction between the unperturbed charge distributions of the (deformed) reactants and is usually attractive. The Pauli repulsion, Δ*E*_Pauli_(ζ), comprises the destabilizing interaction between occupied closed‐shell orbitals of both fragments due to the Pauli principle. The orbital interaction energy, Δ*E*_oi_(ζ), accounts for polarization and charge transfer between the fragments, such as HOMO‐LUMO interactions. A detailed, step‐by‐step, guide on how to perform and interpret the ASM and EDA can be found in Ref. 26 f. Note that the concepts of Pauli repulsion and orbital interaction that feature in our canonical EDA also have been successfully applied to reactions that were studied using other decomposition schemes such as DFT‐SAPT^[29]^ or valence bond (VB) theory.^[30]^

In both the activation strain and energy decomposition diagrams in this study, the energy terms were projected onto the shortest of the two newly forming C⋅⋅⋅C bond between the 1,3‐butadiene and (LA‐)methyl acrylate. This critical reaction coordinate undergoes a well‐defined change during the reaction from the reactant complex via the transition state to the cycloadduct and is shown to be a valid reaction coordinate for studying cycloadditions.[^9^, ^31^]

## Results and Discussion

### Lewis acid‐methyl acrylate binding and trends in reactivity

First, we analyzed the nature and strength of the interaction between methyl acrylate (**MA**) and the various Lewis acids (LAs) across the periodic table, that is, the hydrogen, pnictogen, chalcogen, and halogen bond, using the activation strain model (ASM)^[26]^ of reactivity and energy decomposition analysis (EDA)^[28]^ schemes (Table 1). The binding energies of the LAs become increasingly more stabilizing from pnictogen to chalcogen, halogen, and hydrogen bonds. This trend is exclusively dictated by the interaction energies, which are stabilizing and become increasingly so along this series. Not surprisingly, the inclusion of dispersion in our calculations (ZORA‐BP86‐D3(BJ)/TZ2P level) makes the computed interaction energies slightly more stabilizing (associated with slightly shorter LA⋅⋅⋅O=C distances, see Table S1 in the Supporting Information). Despite that, the above‐commented trend is identical, therefore, validating our selected computational method for the present study (ZORA‐BP86/TZ2P). The corresponding LA⋅⋅⋅O=C distance follows the same trend and becomes systematically shorter. As previously shown in the literature,^[9]^ the interaction consists of both electrostatic interactions and orbital (*i. e*., covalent) interactions, which becomes increasingly more stabilizing along the series. The latter of these two attractive interactions is, as we will show later, of significant importance for the catalytic ability of the Lewis acid. Surprisingly, the ratio between these two types of interactions changes drastically when going from pnictogen to chalcogen to halogen to hydrogen bonds. In the pnictogen bond, the electrostatic interaction is 2.5 times as large as the orbital interactions, indicating the large electrostatic character of this bond. For the halogen bond, on the other hand, both interactions have an equal contribution to the overall halogen bond strength. The binding energy becomes further enhanced when going from Period 3 to Period 4 and Period 5 LAs, due to (i) a lower‐lying σ* acceptor orbital, which, in turn, engages in a more stabilizing (*i. e*., smaller) HOMO‐LUMO gap with **MA** and (ii) the more electropositive nature of the heavier atoms involved in the interaction with **MA**, amplifying the stabilizing electrostatic interactions.

**Table 1:** Energy decomposition analysis terms (in kcal mol^−1^) and LA⋅⋅⋅O=C distance (in Å) computed on LA‐methyl acrylate adducts.^[a]^

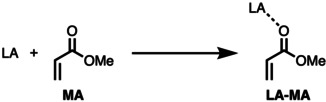							
**LA**	Δ*E*	Δ*E*_strain_	Δ*E*_int_	Δ*V*_elstat_	Δ*E*_Pauli_	Δ*E*_oi_	r(LA⋅⋅⋅O=C)
PF_3_	−1.6	0.2	−1.8	−5.2	5.7	−2.2	3.017
AsF_3_	−5.0	0.6	−5.5	−13.3	14.6	−6.8	2.723
SbF_3_	−7.5	0.9	−8.4	−20.3	22.6	−10.7	2.702
SF_2_	−3.8	0.5	−4.4	−12.2	16.3	−8.5	2.574
SeF_2_	−7.0	1.1	−8.2	−20.6	27.0	−14.5	2.499
TeF_2_	−10.1	1.4	−11.5	−27.3	34.2	−18.4	2.518
ClF	−8.5	1.6	−10.1	−21.5	33.1	−21.6	2.292
BrF	−11.2	1.5	−12.7	−25.3	35.2	−22.5	2.354
IF	−11.6	1.1	−12.7	−26.1	33.4	−20.0	2.505
HF	−10.3	1.0	−11.2	−17.7	19.6	−13.2	1.618

[a] Computed at ZORA‐BP86/TZ2P.

After analyzing the bonding situation in the **LA‐MA** complexes, we focused on the Diels‐Alder reaction of these activated species with **B**. As expected, in all cases, the cycloaddition reaction occurs in a concerted manner through the corresponding six‐membered transition state (see Figure S1 in the Supporting Information). The electronic reaction barriers (Δ*E* ^≠^) and the reaction energies (Δ*E*_rxn_) of the uncatalyzed (**MA**) and LA‐catalyzed DA reaction between **B** and **LA‐MA** are provided in Table 2. Two clear periodic reactivity trends can be observed. In the first place, binding of a LA catalyst significantly accelerates the DA reaction between **B** and **MA**. As expected, the uncatalyzed reaction has the highest reaction barrier (13.2 kcal mol^−1^), which, for Period 3 systematically decreases going from a pnictogen (**PF_3_‐MA**; 12.5 kcal mol^−1^) to chalcogen (**SF_2_‐MA**; 11.7 kcal mol^−1^) to hydrogen (**HF‐MA**; 11.0 kcal mol^−1^) to halogen (**ClF‐MA**; 10.3 kcal mol^−1^) bond‐catalyzed Diels‐Alder reaction. The reactivity trends for the Period 4 and 5 Lewis acids are slightly different, namely, **MA**>**AsF_3_‐MA**>**HF‐MA**>**SeF_2_‐MA**>**BrF‐MA** for Period 4 and **MA**>**HF‐MA**>**SbF_3_‐MA**>**TeF_2_‐MA**>**IF‐MA** for Period 5, because the pnictogen, chalcogen, and halogen bonds involving Period 4 and 5 atoms engage in a stronger donor‐acceptor interaction with **MA**, which is, as we will show later, crucial for the catalytic effect of the Lewis acids, than the hydrogen‐bonded analog. Secondly, the Diels‐Alder reaction between **B** and **LA‐MA** catalyzed by a Period 5 LA goes with a 0.3 to 2.3 kcal mol^−1^ lower barrier than those promoted by their Period 3 analogs. Interestingly, however, all reactions studied in this work, both uncatalyzed and catalyzed, are similarly exergonic. Note that the computed trends in reactivity at ZORA/BP86/TZ2P agree well with those calculated at the dispersion‐corrected ZORA‐BP86‐D3(BJ)/TZ2P level, see Table S2 in the Supporting Information.

**Table 2:** Electronic reaction barriers (Δ*E* ^≠^), reaction energies (Δ*E*_rxn_) (in kcal mol^−1^) for the uncatalyzed and Lewis acid‐catalyzed Diels‐Alder reaction between 1,3‐butadiene (**B**) and methyl acrylate (**LA‐MA**).^[a]^

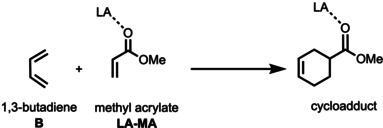		
**LA‐MA**	Δ*E* ^≠^	Δ*E*_rxn_
MA	13.2	−37.0
PF_3_‐MA	12.5	−36.8
AsF_3_‐MA	11.3	−36.6
SbF_3_‐MA	10.2	−36.7
SF_2_‐MA	11.7	−36.5
SeF_2_‐MA	10.1	−37.0
TeF_2_‐MA	10.1	−36.2
ClF‐MA	10.3	−36.2
BrF‐MA	9.9	−36.2
IF‐MA	9.8	−36.3
HF‐MA	11.0	−36.2

[a] Computed at ZORA‐BP86/TZ2P.

### Catalytic effect of weakly interacting Lewis acids

**Figure 1:**
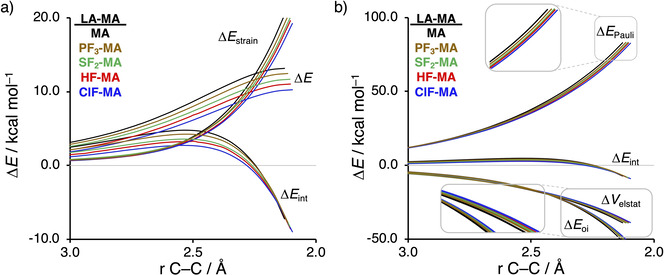
a) Activation strain analyses and b) energy decomposition analyses of the uncatalyzed and LA‐catalyzed Diels‐Alder reaction between **B** and **LA‐MA**, where the energy values are plotted from the reactants to the transition state and projected onto the shorter newly forming C_**B**_⋅⋅⋅C_β_ bond between **B** and **LA‐MA**, computed at ZORA‐BP86/TZ2P.

First, we examine the physical factors leading to the computed periodic reactivity trend upon going, along a period, from the uncatalyzed to the hydrogen, halogen, chalcogen, and pnictogen bond‐catalyzed Diels‐Alder reactions by applying the activation strain model (ASM) of reactivity.^[26]^ To this end, we have analyzed and compared the reactivity trend involving the uncatalyzed (**MA**), and Period 3‐catalyzed (**PF_3_**, **SF_2_**, **HF**, and, **ClF**) DA reactions, which has the largest, and hence clearest, differences along the various catalysts (Figure 1a). Note that the activation strain diagrams (ASDs) of the DA reaction catalyzed by Period 4 and Period 5 Lewis acids can be found in Figures S2 and S3 (Supporting Information). The enhanced reactivity, *i. e*., lower reaction barrier, of the LA‐catalyzed reactions originates predominantly from a less destabilizing strain energy along the entire reaction coordinate. The interaction energy, on the other hand, is for all reactions, in the transition state region, nearly identical and hence not responsible for the observed trend in reactivity.^[32]^

The trend in strain energy, which becomes increasingly less destabilizing from **MA**>**PF_3_‐MA**>**SF_2_‐MA**>**HF‐MA**>**ClF‐MA**, can be explained by looking at the degree of asynchronicity of these reactions (**MA**: Δ*r* ^TS^_C⋅⋅⋅C_=0.38 Å; **PF_3_**‐**MA**: Δ*r* ^TS^_C⋅⋅⋅C_=0.43 Å; **SF_2_**‐**MA**: Δ*r* ^TS^_C⋅⋅⋅C_=0.46 Å; **HF**‐**MA**: Δ*r* ^TS^_C⋅⋅⋅C_=0.48 Å; **ClF**‐**MA**: Δ*r* ^TS^_C⋅⋅⋅C_=0.51 Å, where Δ*r* ^TS^_C⋅⋅⋅C_ refers to the difference between the newly forming C⋅⋅⋅C bond lengths in the TS, see Figure S1). The higher degree of asynchronicity of **HF‐MA** leads to a lower degree of deformation of the reactants since the C_**B**_⋅⋅⋅C_α_ bond forms behind of the C_**B**_⋅⋅⋅C_β_ bond, resulting in a less destabilizing strain energy. To understand why the interaction energy does not play a prominent role in the catalysis of the herein studied reactions, we apply the energy decomposition analysis (EDA) (Figure 1b). Contrary to the commonly accepted view that LAs enhance the orbital interactions of LA‐catalyzed Diels‐Alder reactions,^[14]^ we find that not only the orbital interactions but also the electrostatic interactions, show a trend opposite to the trend in reaction barriers, that is, the uncatalyzed DA reaction has a more stabilizing Δ*E*_oi_ and Δ*V*_elstat_ than the LA‐catalyzed analogs. The Pauli repulsion follows, in line with our previous work on LA‐catalyzed Diels‐Alder, Michael addition, and ring‐opening reactions,^[9]^ the trend in reaction barrier, namely, the uncatalyzed reaction exhibits the most destabilizing Pauli repulsion while the halogen bond mediated reactions has the least destabilizing Pauli repulsion. The reduction of stabilizing orbital and electrostatic interactions and the decrease in destabilizing Pauli repulsion for the LA‐catalyzed DA reactions effectively cancel each other, leading to an interaction energy that is nearly identical to the uncatalyzed DA reaction.^[32]^

**Figure 2:**
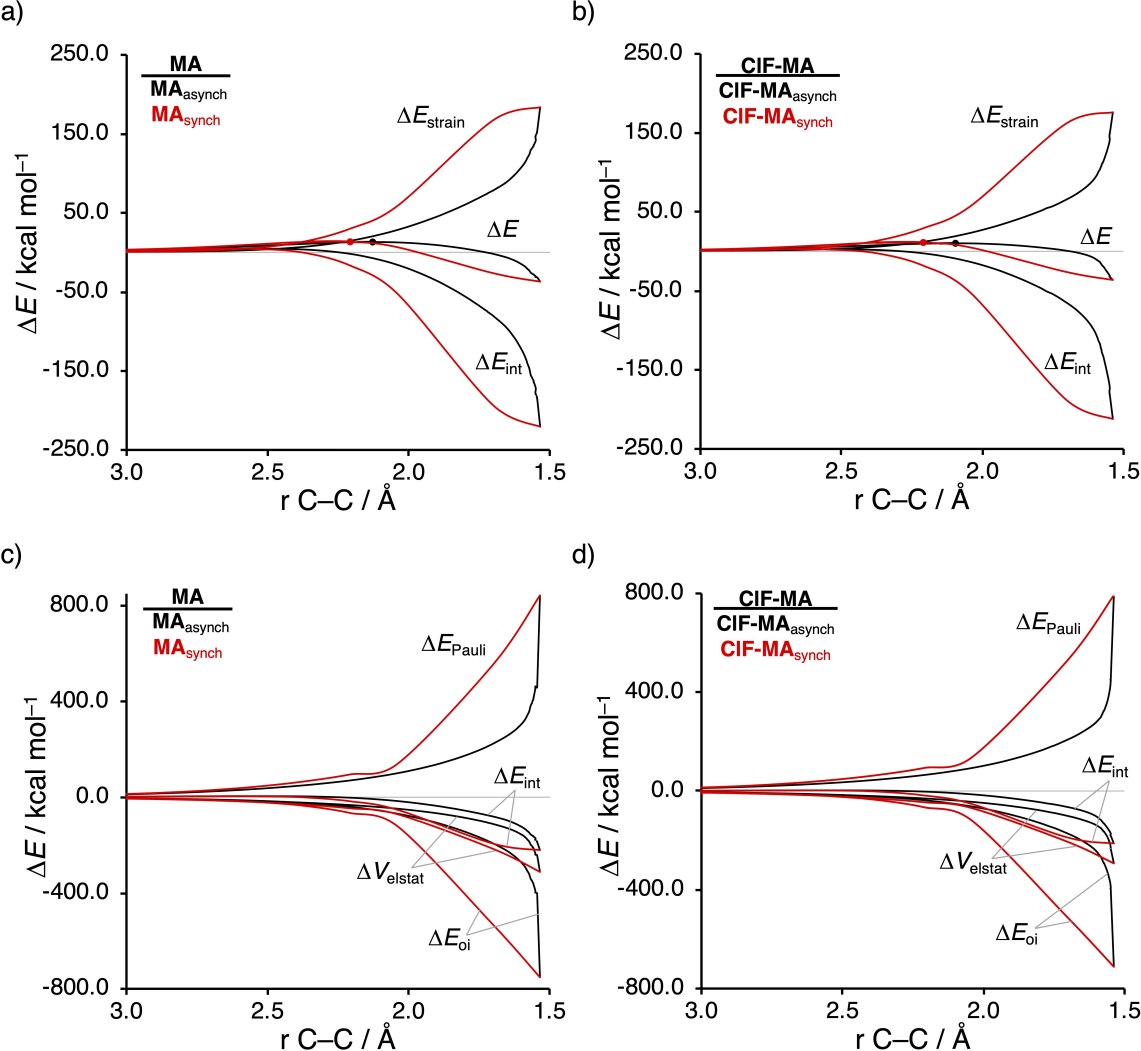
a, b) Activation strain analyses and c, d) energy decomposition analyses of the asynchronous (black) and constraint synchronous (red) Diels‐Alder reactions between **B** and **MA** and **ClF‐MA**, where the transition states are indicated with a dot, the energy values are plotted from the reactants to the cycloadduct and projected onto the newly forming C_**B**_⋅⋅⋅C_β_ bond between **B** and (**ClF‐)MA**, computed at ZORA‐BP86/TZ2P.

As prior discussed, the degree of asynchronicity is the key factor in the reactivity of these Diels‐Alder reactions. We, therefore, want to understand the origin of the asynchronicity of the herein studied reactions, which ultimately results in the catalytic effect of weakly interacting LAs by allowing for less reactant deformation and hence activation strain. To this end, we compare, in analogy with our work on iminium‐catalyzed Diels‐Alder reactions,^[9d]^ the actual concerted asynchronous Diels‐Alder reaction to the analogous process which is artificially constrained to be concerted synchronous. In Figure 2a and 2b, we solely focus on the two extremes, namely, the activation strain diagrams (ASDs) of the asynchronous and synchronous uncatalyzed (**MA**) and **ClF‐**catalyzed DA reaction. The ASDs of all other reactions (**PF_3_‐MA**, **SF_2_‐MA**, **HF‐MA**) show the same characteristics (Figure S5‐S7). Both uncatalyzed and catalyzed synchronous DA reactions proceed with a higher barrier compared to their asynchronous counterpart (ΔΔ*E* ^≠^_**MA**_=0.2 kcal mol^−1^; ΔΔ*E* ^≠^_**ClF‐MA**_=0.9 kcal mol^−1^), even though the synchronous DA reactions experience a more stabilizing interaction energy. The strain energy, on the other hand, is the most destabilizing for the synchronous DA reaction pathway, because both newly forming C⋅⋅⋅C bonds between **B** and (**ClF‐)MA** are formed simultaneously, leading to more deformation and hence more strain since all involved carbon atoms are pyramidalizing at the same time. The strain energies of both the asynchronous and synchronous DA at the product side are eventually identical because the reactions yield the same cycloadduct and are, therefore, deformed to the same extent.

Next, we turn to the energy decomposition analysis (EDA)^[28]^ to get a better understanding of why the interaction energy is more stabilizing for the synchronous compared to the asynchronous DA reaction (Figure 2c and 2d). In contrast with the widely accepted view that the asynchronicity originates from enhanced orbital interactions due to a larger LUMO‐coefficient on the β‐carbon,^[33]^ we find that the asynchronous pathway goes with a less stabilizing orbital interaction. Instead, what drives the system away from synchronicity is a significantly more destabilizing Pauli repulsion in the synchronous reaction mode, originating from a larger occupied‐occupied orbital overlap of the synchronous DA reaction. To reduce this occupied‐occupied overlap and thus the Pauli repulsion, the reaction mode becomes asynchronous, even though this results in the aforementioned loss of the favorable orbital and electrostatic interactions. Thus, it is the interplay between this reduction of destabilizing Pauli repulsion and loss of stabilizing orbital and electrostatic interactions that determine the degree of asynchronicity of a Diels‐Alder reaction. Note that we do find, as previously reported in the literature,^[14]^ a larger 2*p_z_*‐coefficient on the β‐carbon of the **MA** and **Cl‐MA** LUMO than on the α‐carbon (Figure S8). This, however, does not result in a stronger orbital interaction, as prior discussed, because the orbital overlaps of both the normal and inverse electron demand are larger and hence more stabilizing for the synchronous reaction pathway (see Figure S9).

**Figure 3:**
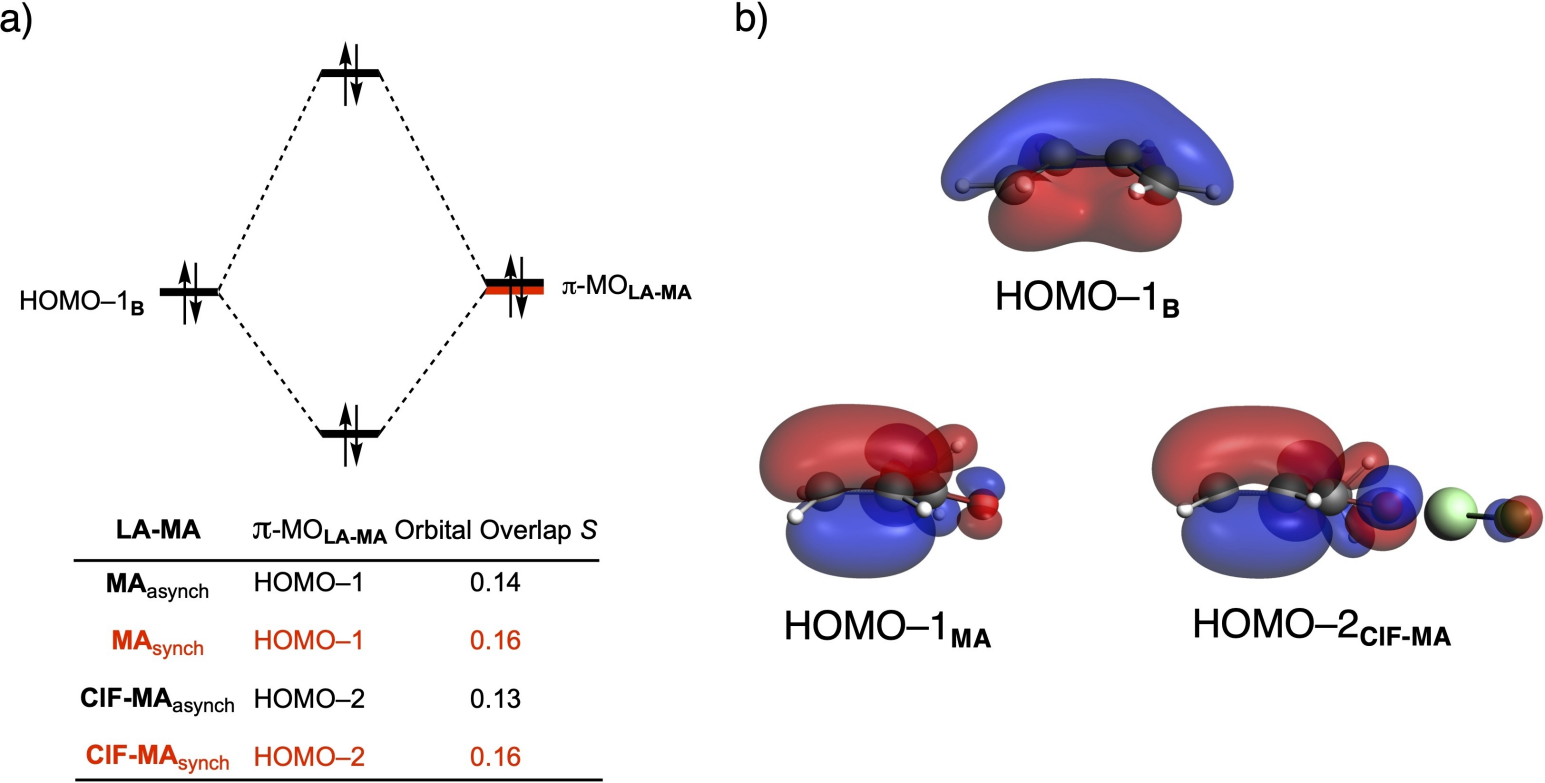
a) Molecular orbital diagram and the most significant occupied orbital overlaps of the asynchronous (black) and constraint synchronous (red) Diels‐Alder reactions between **B** and **MA** and **ClF‐MA**; and b) key occupied orbitals (isovalue=0.03 Bohr^−3/2^) of **B**, **MA** and, **ClF‐MA**, computed on consistent geometries with a C_**B**_⋅⋅⋅C_β_ bond length between **B** and **(ClF‐)MA** of 2.128 Å at ZORA‐BP86/TZ2P.

**Figure 4:**
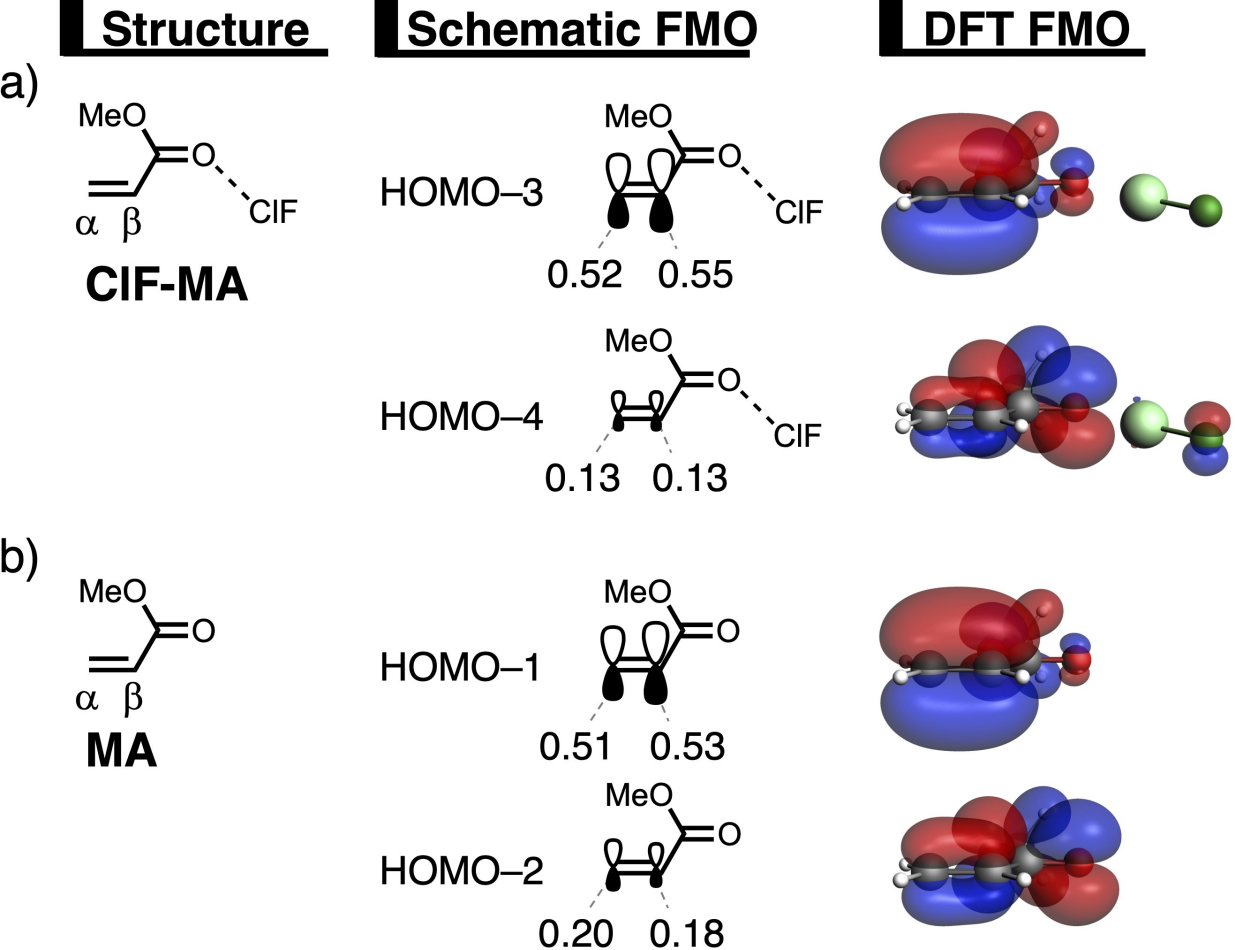
Key occupied π‐MOs (isovalue=0.03 Bohr^−3/2^) computed at the equilibrium structures of a) **ClF‐MA** and b) **MA**, where the MO‐coefficients of the α‐carbon and β‐carbon 2*p*_z_ atomic orbitals, contributing to the occupied orbitals, are shown in the schematic π‐MOs, computed at ZORA‐BP86/TZ2P.

The origin of the less destabilizing Pauli repulsion along the asynchronous reaction pathway was further investigated by performing a Kohn‐Sham molecular orbital (KS‐MO) analysis (Figure 3a).[^28b^, ^34^] The occupied molecular orbitals of **B**, as well as, **MA** and **ClF‐MA** were quantified at consistent geometries with a C_**B**_⋅⋅⋅C_β_ bond length between **B** and **LA‐MA** of 2.128 Å. Performing this analysis at a consistent point along the reaction coordinate (near all transition structures), rather than the transition state alone, ensures that the results are not skewed by the position of the transition state.[^20^, ^26^] The most important occupied orbitals of **LA‐MA** are the HOMO‐1 and HOMO‐2 of **MA** and **ClF‐MA**, respectively, where the 2*p_z_* atomic orbitals located on the C=C double bond are in‐phase. The participating occupied orbital on **B** is the HOMO‐1, where all 2*p_z_* atomic orbitals, located on the reacting C=C double bonds, are in‐phase (Figure 3b). For both **MA** and **ClF‐MA**, the synchronous reaction pathway experiences a larger orbital overlap between the filled orbitals of **B** and **LA‐MA** compared to the asynchronous reaction mode (*S*_**MA**synch_=0.16 and *S*_**MA**asynch_=0.14; *S*_**ClF‐MA**synch_=0.16 and *S*_**ClF‐MA**asynch_=0.13). We can trace the larger orbital overlap and, consequently, more destabilizing Pauli repulsion, for the synchronous DA reaction back to the asymmetry in the MO‐coefficients of the 2*p_z_* atomic orbitals on the α‐ and β‐carbon atoms of **LA‐MA** (Figure 4). The MO‐coefficient of the 2*p_z_* atomic orbital on the α‐carbon of HOMO‐1 and HOMO‐3 of **MA** and **ClF‐MA**,^[35]^ respectively, lead to a larger orbital overlap and, therefore, more Pauli repulsion with the filled orbital of the incoming **B** than the β‐carbon, which has a smaller MO‐coefficient and hence less orbital overlap, *i. e*., less Pauli repulsion, with **B**. To avoid the otherwise more destabilizing Pauli repulsion of **B** with the α‐carbon of **LA‐MA**, the formation of the new C_**B**_⋅⋅⋅C_α_ bond between **LA‐MA** and **B** lags behind the formation of the C_**B**_⋅⋅⋅C_β_ bond, resulting in an asynchronous DA reaction.

In addition, we want to understand why the DA reaction involving **ClF‐MA** is significantly more asynchronous than **MA** (**ClF‐MA**: Δr^TS^_C⋅⋅⋅C_=0.52 Å; **MA**: Δr^TS^_C⋅⋅⋅C_=0.38 Å), which, ultimately, is the driving force behind the catalytic effect of the herein studied LAs on the Diels‐Alder reaction (*vide supra*). In order to understand this difference, we need to compare the MO‐coefficients of the 2*p_z_* atomic orbitals on the α‐ and β‐carbon of the key π‐MOs of **ClF‐MA** and **MA** (Figure 4). As prior discussed, the HOMO‐3 of **ClF‐MA** has a larger MO‐coefficient on the α‐ than on the β‐carbon. In addition, the MO‐coefficients on the α‐ and β‐carbon of the HOMO‐4 are identical, due to a strong donor‐acceptor interaction between **ClF** and **MA** (Δ*E*_oi,**ClF‐MA**_=−21.6 kcal mol^−1^), which effectively polarizes the π‐density away from the C=C double bond equalizing the originally asymmetric π‐orbital of **MA** (*vide infra*). Thus, the net effect of both occupied orbitals works in favor of an asynchronous reaction mode. On the contrary, the MO‐ coefficients of the key HOMOs of **MA** do not both point towards the observed asynchronous reaction mode. In line with **ClF‐MA**, the MO‐coefficient of the α‐carbon of HOMO‐1_**MA**_ is larger than on the β‐carbon, driving the reaction towards the observed asynchronous reaction mode. But, this effect gets partially countered by the asymmetry in the MO‐coefficients of the HOMO‐2_**MA**_, which has a larger orbital amplitude on the β‐carbon than on the α‐carbon, resulting in a DA reaction that has a smaller degree of asynchronicity and hence a higher reaction barrier than **ClF‐MA**.

**Figure 5:**
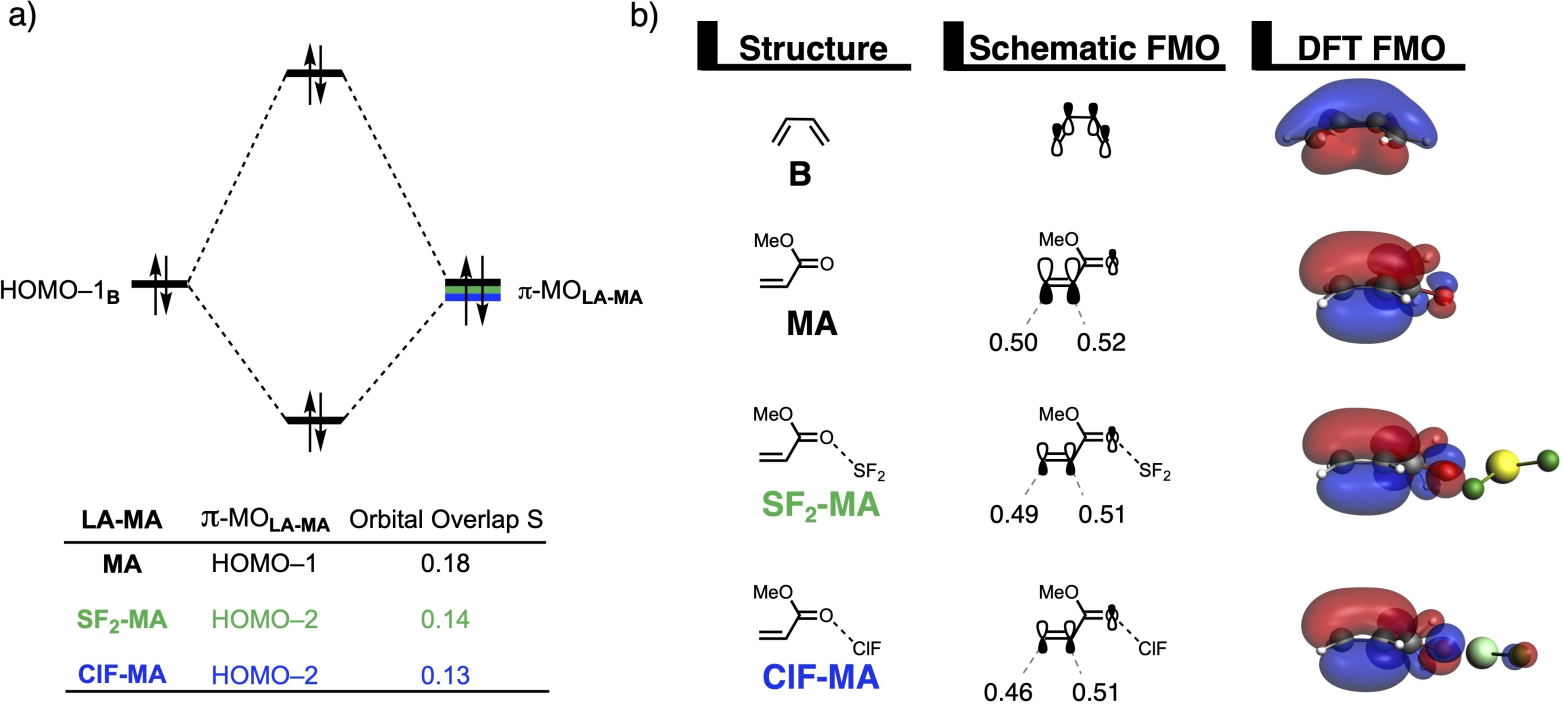
a) Molecular orbital diagram and the most significant occupied orbital overlaps of the uncatalyzed and LA‐catalyzed Diels‐Alder reaction between **B** and **LA‐MA** and b) key occupied orbitals (isovalue=0.03 Bohr^−3/2^) where the MO‐coefficients of the α‐carbon and β‐carbon 2*p*_z_ atomic orbitals, contributing to the occupied orbitals, are shown in the schematic π‐MOs, computed on consistent geometries with a C_**B**_⋅⋅⋅C_β_ bond length between **B** and **LA‐MA** of 2.128 Å at ZORA‐BP86/TZ2P.

After having established the origin of the different degrees of asynchronicity and, thus, the reason why hydrogen, halogen, chalcogen, and pnictogen bonds are able to catalyze Diels‐Alder reactions, we want to understand why these Lewis acids are able to reduce the Pauli repulsion (Figure 1b). The less destabilizing Pauli repulsion for the LA‐catalyzed Diels‐Alder reaction derives from the reduced occupied‐occupied orbital overlap between **B** and **LA‐MA**. The occupied molecular orbitals of **B** and **LA‐MA** were quantified at consistent geometries with a C_**B**_⋅⋅⋅C_β_ bond length between **B** and the dienophile of 2.128 Å (Figure 5). The most important occupied π‐MOs of **LA‐MA** involved in the two‐center four‐electron interaction are the HOMO‐1 of **MA** and the HOMO‐2 of **ClF‐MA** and **SF_2_‐MA**. Notably, the π‐MOs of **LA‐MA** are the same π‐orbital located on the C=C double bond of the dienophile. The contributing occupied orbital of **B** is the HOMO‐1, where all 2*p_z_* AOs located on both reacting C=C double bonds are in‐phase. The orbital overlap between the HOMO‐1_**B**_ and the occupied π‐MO_**LA‐MA**_ of the dienophile is the largest and, therefore, most destabilizing (*S*=0.18) for **MA** (uncatalyzed reaction) and smallest and least destabilizing for **ClF‐MA** (*S*=0.13) (Figure 5a). Binding of a Lewis acid to **MA** polarizes, due to the strong donor‐acceptor interaction (Table 1), the occupied π‐MO_**LA‐MA**_ located on the C=C double bond of the dienophile away from the incoming **B** (Figure 5b), which, in turn, decreases the occupied‐occupied orbital overlap and hence the Pauli repulsion. In addition, the above‐discussed larger degree of asynchronicity also plays a role in the reduction of the occupied‐occupied orbital overlap. A more asynchronous reaction, *i. e*., the DA reaction catalyzed by a LA, has, due to the longer C_**B**_⋅⋅⋅C_α_ bond, less orbital overlap at the α‐carbon of **LA‐MA**, manifesting in less Pauli repulsion between **B** and **LA‐MA**. We have observed these exact phenomena also in our analysis of Lewis acid and iminium ion‐catalyzed Diels‐Alder, Michael addition, and ring‐opening reactions,^[9]^ which again demonstrates that the concept of *Pauli repulsion‐lowering catalysis* is a broader phenomenon and independent of how the Lewis acid is bound to the dienophile.

**Figure 6:**
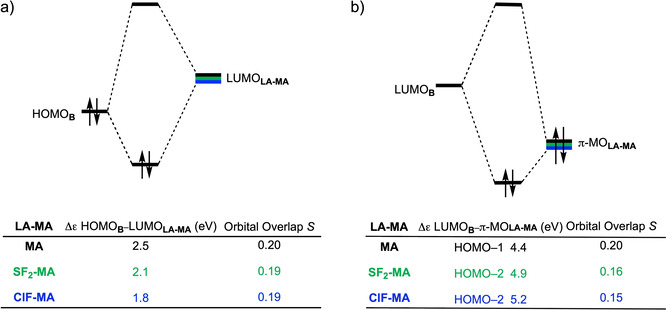
Molecular orbital diagrams with orbital energies and overlaps for a) normal electron demand (NED) HOMO_**B**_‐**LUMO_LA‐MA_** interactions; and for b) inverse electron demand (IED) LUMO_**B**_ −π‐MO_**LA‐MA**_ interactions of the uncatalyzed and LA‐catalyzed Diels‐Alder reaction between **B** and **LA‐MA**, computed on consistent geometries with a C_**B**_⋅⋅⋅C_β_ bond length between **B** and **LA‐MA** of 2.128 Å at ZORA‐BP86/TZ2P.

At last, we address why the orbital interactions for the uncatalyzed DA reaction are more stabilizing than for the LA‐catalyzed counterpart, which effectively compensates the observed trend in Pauli repulsion and hence results in a nearly identical interaction energy for the uncatalyzed and LA‐catalyzed reactions (Figure 1). In line with the current textbook rationale,^[14]^ binding a Lewis acid to **MA** strengthens the normal electron demand (NED) orbital interaction by lowering the LUMO_**LA‐MA**_ energy, but, simultaneously, the LA catalyst suppresses the inverse electron demand (IED) orbital interaction since it also lowers the π‐MO_**LA‐MA**_ and hence increases the LUMO_**B**_ ‐π‐MO_**LA‐MA**_ energy gap. The latter effect overrules the former, resulting in orbital interactions for the LA‐catalyzed reaction between **B** and **LA‐MA** which are less favorable than the uncatalyzed analog. By performing a Kohn‐Sham molecular orbital (KS‐MO) analysis on consistent geometries with a C_**B**_⋅⋅⋅C_β_ bond length between **B** and **LA‐MA** of 2.128 Å,[^28b^, ^34^] we found that the NED orbital energy gap between HOMO_**B**_‐LUMO_**LA‐MA**_ decreases from 2.5 eV for the uncatalyzed reaction to 1.8 eV for the **ClF**‐catalyzed reaction (Figure 6a). This reduction of the orbital energy gap is significant enough to overcome the minor loss of orbital overlap, which results from the more asynchronous reaction mode, and hence results in a more favorable NED interaction. However, binding of a LA stabilizes all orbitals of **LA‐MA**, thus also the key occupied π‐MO_**LA‐MA**_, which effectively results in an enlargement of the IED LUMO_**B**_ ‐π‐MO_**LA‐MA**_ energy gap going from 4.4 eV for the uncatalyzed reaction to 5.2 eV for the **ClF**‐catalyzed reaction (Figure 6b). This, in conjunction with a significantly reduced orbital overlap as a result of the more asynchronous reaction mode, leads to a weaker IED interaction for the LA‐catalyzed compared to the uncatalyzed DA reaction.

### Transitioning from Period 3 to Period 5‐based Lewis acid catalysts

**Figure 7:**
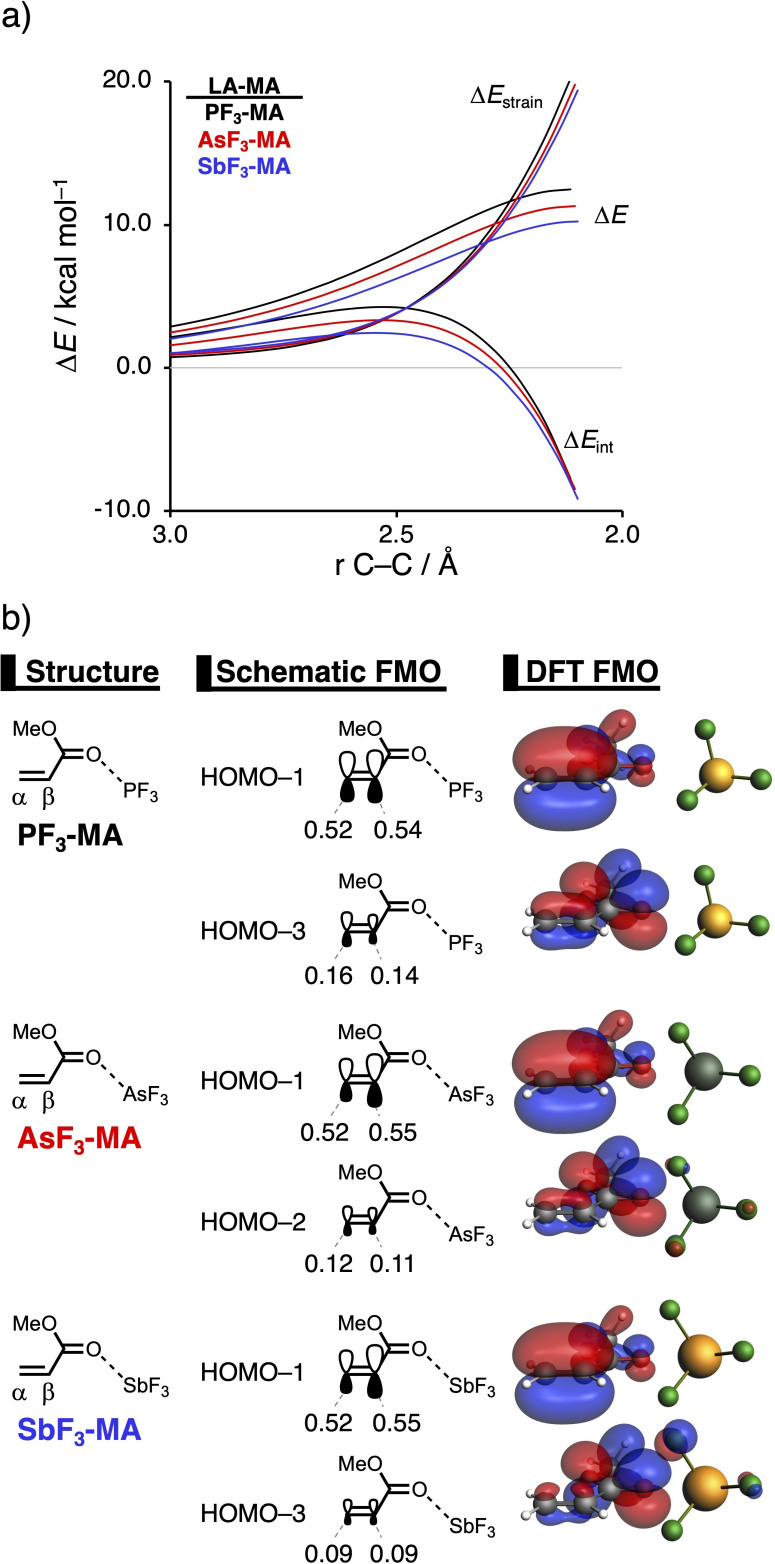
a) Activation strain analyses of the **PF_3_**‐, **AsF_3_**‐,and **SbF_3_**‐catalyzed Diels‐Alder reactions between **B** and **LA‐MA**, where the energy values are projected onto the shorter newly forming C_**B**_⋅⋅⋅C_β_ bond between **B** and **LA‐MA**; b) Key occupied π‐MOs (isovalue=0.03 Bohr^−3/2^) computed at the equilibrium structures of **PF_3_‐MA**, **AsF_3_‐MA**, and **SbF_2_‐MA**, where the MO‐coefficients of the α‐carbon and β‐carbon 2*p*_z_ atomic orbitals, contributing to the occupied orbitals, are shown in the schematic π‐MOs, computed at ZORA‐BP86/TZ2P.

In the next section, we want to establish the periodic reactivity trend upon descending a group, that is, why Period 5‐based LAs accelerate the Diels‐Alder (DA) reaction between **B** and **LA‐MA** to a larger extent than their Period 3‐based analogs. Figure 7a shows the activation strain diagrams (ASDs) from the separate reactants to the transition states for the Diels‐Alder reaction between **B** and **LA‐MA** catalyzed by the Group 15 LAs: **PF_3_**, **AsF_3_**, and **SbF_3_** of Periods 3, 4, and 5, respectively. The DA reaction catalyzed by the Group 16 and Group 17 LAs show the same, but less pronounced characteristics (Figure S10 and S11). The enhanced reactivity of the DA reaction catalyzed by the Period 5 LA **SbF_3_** originates exclusively from a less destabilizing strain energy. On the other hand, the interaction energy is, in the transition state region, identical and hence not responsible for the observed trend in reaction barriers.

The less destabilizing strain energy for **SbF_3_‐MA** can be ascribed to the larger degree of asynchronicity compared to **PF_3_‐MA** and **AsF_3_‐MA** (**PF_3_**‐**MA**: Δ*r* ^TS^_C**⋅⋅⋅**C_=0.43; **AsF_3_**‐**MA**: Δ*r* ^TS^_C⋅⋅⋅C_=0.47; **SbF_3_**‐**MA**: Δ*r* ^TS^_C⋅⋅⋅C_=0.52), which leads to a lower degree of deformation of the reactants, **B** and **LA‐MA**, since the C_**B**_⋅⋅⋅C_β_ bond forms ahead of the C_**B**_⋅⋅⋅C_α_. This difference in the degree of asynchronicity can again be explained by looking at the MO‐coefficients of the 2*p_z_* atomic orbitals on the α‐ and β‐carbon of the key π‐MOs of **PF_3_‐MA**, **AsF_3_‐MA**, and **SbF_3_‐MA** (Figure 7b). The HOMO‐1 of **PF_3_‐MA** has a larger MO‐coefficient on the α‐ than on the β‐carbon inducing an asynchronous reaction mode. However, this effect gets partly compensated by the HOMO‐3 of **PF_2_‐MA**, because it shows the opposite characteristics, that is, a smaller MO‐coefficient on the α‐carbon compared to the β‐carbon, making the reaction moderately asynchronous. The same effect, only less pronounced due to a more asymmetric HOMO‐1, can also be seen for the **AsF_3_**‐**LA**, making this reaction slightly more asynchronous. The reaction involving **SbF_3_‐MA**, on the contrary, follows a more asynchronous reaction mode, due to (i) a larger asymmetry in the MO‐coefficients of the HOMO‐1 compared to **PF_3_‐MA**; and (ii) a symmetric HOMO‐3 which does not counter the asynchronicity induced by the HOMO‐1. Both of these effects are initiated by the stronger donor‐acceptor interaction between **SbF_2_** and **MA** compared to the interaction between **PF_3_** or **AsF_3_** and **MA** (Table 1; Δ*E* ${}_{\mathrm{oi},PF{}_{3}\text{-}MA}$ =−2.2 kcal mol^−1^, Δ*E* ${}_{\mathrm{oi},AsF{}_{3}\text{-}MA}$ =−6.8 kcal mol^−1^, and Δ*E* ${}_{\mathrm{oi},SbF{}_{3}\text{-}MA}$ =−10.7 kcal mol^−1^), which results in a more significant polarization of the π‐MOs away from the C=C double bond of **SbF_3_‐MA**, amplifying the asymmetry in the MO‐coefficients of the 2*p_z_* atomic orbitals on the α‐ and β‐carbon atoms of **SbF_3_‐MA** and hence making the reaction more asynchronous.

## Conclusion

Our study quantifies and pinpoints the catalytic effect of various weakly interacting Lewis acids, across the periodic table, on the Diels‐Alder (DA) reaction between 1,3‐butadiene (**B**) and methyl acrylate (**MA**). Lewis acids (LAs), binding via hydrogen, halogen, chalcogen, and pnictogen bonds, efficiently catalyze the DA reaction by lowering the reaction barrier up to 3 kcal mol^−1^. This catalytic effect decreases going, along a period, from halogen to hydrogen to chalcogen to pnictogen‐bonded Lewis acids. In addition, the Period 5‐based Lewis acids catalyze the DA reaction to a larger extent than the corresponding Period 3 and Period 4‐based counterparts.

Our activation strain and Kohn‐Sham molecular orbital analyses uncovered that the enhanced reactivity of the Lewis acid‐catalyzed compared to the uncatalyzed DA reaction originates from a larger degree of asynchronicity, which yields a less destabilizing activation strain since one newly forming C⋅⋅⋅C bond forms later than the other. The asynchronicity of these DA reactions are caused by the asymmetry in the MO‐coefficients of the 2*p_z_* atomic orbitals on the α‐ and β‐carbon of the occupied π‐orbitals of the dienophile, namely, the MO coefficient on the α‐carbon is larger than on the β‐carbon, inducing more occupied‐occupied orbital overlap between **B** and the α‐carbon of **LA‐MA**. To circumvent this repulsive interaction, the formation of the new C_**B**_⋅⋅⋅C_α_ bond between **LA‐MA** and **B** lags behind that of the C_**B**_⋅⋅⋅C_β_ bond, resulting in an asynchronous DA reaction. The stronger the Lewis acid interacts with the dienophile, going from halogen to hydrogen to chalcogen to pnictogen bonds, the larger the asymmetry in the MO‐coefficients of the occupied π‐orbitals of the dienophile, which leads to a more asynchronous reaction mode and hence less repulsion between the reactants and a lower reaction barrier.

The increased rate enhancement upon descending a group from Period 3 to Period 5 is exclusively caused by the reduced activation strain, originating from the increased degree of a synchronicity. The Period 5 LA catalysts enter in a stronger donor‐acceptor interaction with **MA**, due to the higher polarizability and more electropositive nature of the heavier atoms of the LA involved in this interaction. Consequently, the asymmetry in the MO coefficients of the occupied π‐orbitals of the dienophile is more prominent for the Period 5 LAs, making the reaction more asynchronous. The work presented herein demonstrates, once again, the more general applicability of the *Pauli repulsion‐lowering catalysis* concept. Despite that, and different to our previous works on related catalyzed processes,^[9]^ the Pauli repulsion lowering induced by these weakly bonded catalysts is not translated into a stronger interaction between the deformed reactants but is mainly manifested in a more asynchronous reaction mode, which constitutes the causal actor behind the catalytic effect of these Lewis acids in the considered DA reactions.

## Conflict of interest

The authors declare no conflict of interest.

## Supporting information

As a service to our authors and readers, this journal provides supporting information supplied by the authors. Such materials are peer reviewed and may be re‐organized for online delivery, but are not copy‐edited or typeset. Technical support issues arising from supporting information (other than missing files) should be addressed to the authors.

Supporting Information

## References

[1a] F. Fringuelli, A. Taticchi, The Diels-Alder Reaction: Selected Practical Methods, Wiley, Hoboken, 2002.; See also:

[1b] S. Sankararaman, Pericyclic Reaction-A Textbook: Reactions, Applications and Theory, Wiley-VCH, Weinheim, 2005. For a recent review, see:;

[1c] K. N. Houk, F. Liu, Z. Yang, J. I. Seeman, Angew. Chem. Int. Ed. 2021, 10.1002/anie.202001654;

[2] O. Diels, K. Alder, Justus Liebigs Ann. Chem. 1928, 460, 98.

[3] For reviews on the application of DA reactions in total synthesis, see:

[3a] W. Carruthers, Cycloaddition Reactions in Organic Synthesis, Pergamon Press, Oxford, U. K., 1990;

[3b] K.-i. Takao, R. Munakata, K.-i. Tadano, Chem. Rev. 2005, 105, 4779; 16351062 10.1021/cr040632u

[3c] M. Juhl, D. Tanner, Chem. Soc. Rev. 2009, 38, 2983; 19847335 10.1039/b816703f

[3d] K. C. Nicolaou, S. A. Snyder, T. Montagnon, G. Vassilikogiannakis, Angew. Chem. Int. Ed. 2002, 41, 1668; 10.1002/1521-3773(20020517)41:10<1668::aid-anie1668>3.0.co;2-z 19750686

[3e] H. B. Kagan, O. Riant, Chem. Rev. 1992, 92, 1007;

[3f] C. O. Kappe, S. S. Murphree, A. Padwa, Tetrahedron 1997, 53, 14179;

[3g] J.-A. Funel, S. Abele, Angew. Chem. Int. Ed. 2013, 52, 3822; 10.1002/anie.201201636 23447554

[4a] U. Pindur, G. Lutz, C. Otto, Chem. Rev. 1993, 93, 741;

[4b] F. Fringuelli, O. Piermatti, F. Pizzo, L. Vaccaro, Eur. J. Org. Chem. 2001, 439. 10.1021/jo034956e 14629145

[5a] J.-H. Zhou, B. Jiang, F.-F. Meng, Y.-H. Xu, T.-P. Loh, Org. Lett. 2015, 17, 4432; 26351987 10.1021/acs.orglett.5b02066

[5b] D. Yepes, P. Pérez, P. Jaque, I. Fernández, Org. Chem. Front. 2017, 4, 1390;

[5c] M. Bakos, Z. Dobi, D. Fegyverneki, Á. Gyömöre, I. Fernández, T. Soós, ACS Sustainable Chem. Eng. 2018, 6, 10869.

[6a] Y. Kishi, M. Aratani, T. Fukuyama, F. Nakatsubo, T. Goto, S. Inoue, S. Sugiura, H. Kakoi, J. Am. Chem. Soc. 1972, 94, 9217; 4642370 10.1021/ja00781a038

[6b] Y. Kishi, T. Fukuyama, M. Aratani, F. Nakatsubo, T. Goto, S. Inoue, S. Sugiura, H. Kakoi, J. Am. Chem. Soc. 1972, 94, 9219; 4642371 10.1021/ja00781a039

[6c] Y. Kishi, F. Nakatsubo, M. Aratani, T. Goto, S. Inoue, H. Kakoi, S. Sugiura, Tetrahedron Lett. 1970, 11, 5127. 10.1016/s0040-4039(00)96956-9 5501331

[7] Y. Huang, V. H. Rawal, J. Am. Chem. Soc. 2002, 124, 9662. 12175197 10.1021/ja0267627

[8a] A. Wittkopp, P. R. Schreiner, Chem. Eur. J. 2003, 9, 407;12532289

[8b] P. R. Schreiner, Chem. Soc. Rev. 2003, 32, 289; 14518182 10.1039/b107298f

[8c] C. S. Kramer, S. Bräse, Beilstein J. Org. Chem. 2013, 9, 1414. 23946836 10.3762/bjoc.9.158 PMC3740680

[9a] P. Vermeeren, T. A. Hamlin, F. M. Bickelhaupt, I. Fernández, Chem. Eur. J. 2021, 27, 5180; 33169912 10.1002/chem.202004496 PMC8049058

[9b] P. Vermeeren, F. Brinkhuis, T. A. Hamlin, F. M. Bickelhaupt, Chem. Asian J. 2020, 15, 1167; 32012430 10.1002/asia.202000009 PMC7187256

[9c] P. Vermeeren, T. A. Hamlin, I. Fernández, F. M. Bickelhaupt, Angew. Chem. Int. Ed. 2020, 59, 6201; 10.1002/anie.201914582 PMC7187354 31944503

[9d] P. Vermeeren, T. A. Hamlin, I. Fernández, F. M. Bickelhaupt, Chem. Sci. 2020, 11, 8105; 34094173 10.1039/d0sc02901g PMC8163289

[9e] T. A. Hamlin, I. Fernández, F. M. Bickelhaupt, Angew. Chem. Int. Ed. 2019, 58, 8922; 10.1002/anie.201903196 PMC6617756 31033118

[9f] T. Hansen, P. Vermeeren, R. Yoshisada, D. V. Filippov, G. A. van der Marel, J. D. C. Codée, T. A. Hamlin, J. Org. Chem. 2021, 86, 3565. For a recent account on Pauli repulsion-lowering catalysis, see:33538169 10.1021/acs.joc.0c02955 PMC7901664

[9g] T. A. Hamlin, F. M. Bickelhaupt, I. Fernández, Acc. Chem. Res. 2021 10.1021/acs.accounts.1c00016. 33759502

[10] For pnictogen bond catalysis, see for instance:

[10a] F. Yaghoobi, M. Sohrabi-Mahboub, J. Phys. Chem. A 2018, 122, 2781; 29489368 10.1021/acs.jpca.7b12400

[10b] J. Schmauck, M. Breugst, Org. Biomol. Chem. 2017, 15, 8037. 28770945 10.1039/c7ob01599b

[11] For chalcogen bond catalysis, see for instance:

[11a] J. Bamberger, F. Ostler, O. G. Mancheño, ChemCatChem 2019, 11, 5198; 31894187 10.1002/cctc.201901215 PMC6919929

[11b] P. Wonner, L. Vogel, M. Düser, L. Gomes, F. Kniep, B. Mallick, D. B. Werz, S. M. Huber, Angew. Chem. Int. Ed. 2017, 56, 12009; 10.1002/anie.201704816 PMC5638094 28605080

[11c] S. Benz, J. López-Andarias, J. Mareda, N. Sakai, S. Matile, Angew. Chem. Int. Ed. 2017, 56, 812; 10.1002/anie.201611019 27981727

[12] For halogen bond catalysis, see for instance:

[12a] V. de P. N. Nziko, S. Scheiner, J. Org. Chem. 2016, 81, 2589; 26907727 10.1021/acs.joc.6b00344

[12b] Y. Takeda, D. Hisakuni, C.-H. Lin, S. Minakata, Org. Lett. 2015, 17, 318; 25551775 10.1021/ol503426f

[12c] C. W. Kee, M. W. Wong, J. Org. Chem. 2016, 81, 7459; 27486786 10.1021/acs.joc.6b01147

[12d] L. C. Dias, J. Braz. Chem. Soc. 1997, 8, 289;

[12e] S. H. Jungbauer, S. M. Walter, S. Schindler, L. Rout, F. Kniep, S. M. Huber, Chem. Commun. 2014, 50, 6281. 10.1039/c4cc03124e 24796408

[13] For a general overview on weak interaction catalysis, see:

[13a] M. Breugst, J. J. Koenig, Eur. J. Org. Chem. 2020, 5473;

[13b] S. Benz, A. I. Poblador-Bahamonde, N. Low-Ders, S. Matile, Angew. Chem. Int. Ed. 2018, 57, 5408; 10.1002/anie.201801452 PMC5947745 29558562

[14a] I. Fleming, Frontier Orbitals and Organic Chemical Reactions, Wiley, New York, 1976. New edition: I. Fleming, *Molecular Orbitals and Organic Chemical Reactions*, Wiley, Hoboken, **2009**;

[14b] K. N. Houk, Acc. Chem. Res. 1975, 8, 361;

[14c] M. J. R. Dewar, Molecular Orbital Theory for Organic Chemists, Prentice-Hall, Englewood Cliffs, New Jersey, 1975;

[14d] W. T. Borden, Modern Molecular Orbital Theory for Organic Chemists, Prentice-Hall, Englewood Cliffs, New Jersey, 1975.

[15] I. Fernández, F. M. Bickelhaupt, J. Comput. Chem. 2014, 35, 371. 24449044 10.1002/jcc.23500

[16a] G. te Velde, F. M. Bickelhaupt, E. J. Baerends, C. Fonseca Guerra, S. J. A. van Gisbergen, J. G. Snijders, T. Ziegler, J. Comput. Chem. 2001, 22, 931;

[16b] C. Fonseca Guerra, J. G. Snijders, G. te Velde, E. J. Baerends, Theor. Chem. Acc. 1998, 99, 391;

[16c] ADF2018.104, SCM Theoretical Chemistry, Vrije Universiteit: Amsterdam (Netherlands). http://www.scm.com.

[17] A. D. Becke, Phys. Rev. A 1988, 38, 3098. 10.1103/physreva.38.3098 9900728

[18] E. van Lenthe, E. J. Baerends, J. Chem. Phys. 2003, 24, 1142. 10.1002/jcc.10255 12759913

[19a] E. van Lenthe, E. J. Baerends, J. G. Snijders, J. Chem. Phys. 1993, 99, 4597;

[19b] E. van Lenthe, E. J. Baerends, J. G. Snijders, J. Chem. Phys. 1994, 101, 9783;

[19c] E. van Lenthe, A. Ehlers, E. J. Baerends, J. Chem. Phys. 1999, 110, 8943.

[20] T. A. Hamlin, D. Svatunek, S. Yu, L. Ridder, I. Infante, L. Visscher, F. M. Bickelhaupt, Eur. J. Org. Chem. 2019, 378.

[21a] M. Franchini, P. H. T. Philipsen, E. van Lenthe, L. Visscher, J. Chem. Theory Comput. 2014, 10, 1994; 26580526 10.1021/ct500172n

[21b] M. Franchini, P. H. T. Philipsen, L. Visscher, J. Comput. Chem. 2013, 34, 1819. 23720371 10.1002/jcc.23323

[22a] S. Grimme, J. Antony, S. Ehrlich, H. Krieg, J. Chem. Phys. 2010, 132, 154104; 20423165 10.1063/1.3382344

[22b] S. Grimme, S. Ehrlich, L. Goerigk, J. Comput. Chem. 2011, 32, 1456; 21370243 10.1002/jcc.21759

[22c] B. G. Johnson, P. M. W. Gill, J. A. Pople, J. Chem. Phys. 1993, 98, 5612;

[22d] T. V. Russo, R. L. Martin, P. J. Hay, J. Chem. Phys. 1994, 101, 7729.

[23a] K. Fukui, Acc. Chem. Res. 1981, 14, 363;

[23b] L. Deng, T. Ziegler, L. Fan, J. Chem. Phys. 1993, 99, 3823;

[23c] L. Deng, T. Ziegler, Int. J. Quantum Chem. 1994, 52, 731.

[24] X. Sun, T. M. Soini, J. Poater, T. A. Hamlin, F. M. Bickelhaupt, J. Comput. Chem. 2019, 40, 2227. 31165500 10.1002/jcc.25871 PMC6771738

[25] CYLview20; Legault, C. Y., Université de Sherbrooke, 2020 (http://www.cylview.org).

[26a] F. M. Bickelhaupt, J. Comb. Chem. 1999, 20, 114;

[26b] W.-J. van Zeist, F. M. Bickelhaupt, Org. Biomol. Chem. 2010, 8, 3118; 20490400 10.1039/b926828f

[26c] I. Fernández, F. M. Bickelhaupt, Chem. Soc. Rev. 2014, 43, 4953; 24699791 10.1039/c4cs00055b

[26d] L. P. Wolters, F. M. Bickelhaupt, WIREs Comput. Mol. Sci. 2015, 5, 324; 10.1002/wcms.1221 PMC4696410 26753009

[26e] F. M. Bickelhaupt, K. N. Houk, Angew. Chem. Int. Ed. 2017, 56, 10070; 10.1002/anie.201701486 PMC5601271 28447369

[26f] P. Vermeeren, S. C. C. van der Lubbe, C. Fonseca Guerra, F. M. Bickelhaupt, T. A. Hamlin, Nat. Protoc. 2020, 15, 649. 31925400 10.1038/s41596-019-0265-0

[27a] D. H. Ess, K. Houk, J. Am. Chem. Soc. 2007, 129, 10646; 17685614 10.1021/ja0734086

[27b] D. H. Ess, K. Houk, J. Am. Chem. Soc. 2008, 130, 10187. 18613669 10.1021/ja800009z

[28a] F. M. Bickelhaupt, E. J. Baerends, Reviews in Computational Chemistry, (Eds.: K. B. Lipkowitz, D. B. Boyd), Wiley, Hoboken, 2000;

[28b] R. van Meer, O. V. Gritsenko, E. J. Baerends, J. Chem. Theory Comput. 2014, 10, 4432; 26588140 10.1021/ct500727c

[28c] T. A. Hamlin, P. Vermeeren, C. Fonseca Guerra, F. M. Bickelhaupt, Complementary Bonding Analysis, (Eds.: S. Grabowsky), De Gruyter, Berlin, 2021, pp 199–212.

[29a] O. A. Stasyuk, R. Sedlak, C. Fonseca Guerra, P. Hobza, J. Chem. Theory Comput. 2018, 14, 3440; 29926727 10.1021/acs.jctc.8b00034

[29b] B. Jeziorski, R. Moszynski, K. Szalewicz, Chem. Rev. 1994, 94, 1887.

[30] S. S. Shaik, P. C. Hiberty, A Chemist's Guide to Valence Bond Theory. Wiley, Hoboken, 2007.

[31a] I. Fernández, Chem. Sci. 2020, 11, 3769; 34122846 10.1039/d0sc00222d PMC8152634

[31b] I. Fernández, Phys. Chem. Chem. Phys. 2014, 16, 7662. 24638229 10.1039/c4cp00346b

[32] The same general conclusions are obtained at ZORA-BP86/TZ2P and at ZORA-BP86-D3(BJ), see Figure S4 in the Supporting Information.

[33a] R. J. Loncharich, F. K. Brown, K. N. Houk, J. Org. Chem. 1989, 54, 1129;

[33b] D. M. Birney, K. N. Houk, J. Am. Chem. Soc. 1990, 112, 4127;

[33c] B. R. Beno, K. N. Houk, D. A. Singleton, J. Am. Chem. Soc. 1996, 118, 9984;

[33d] A. M. Sarotti, Org. Biomol. Chem. 2014, 12, 187. 24085334 10.1039/c3ob41628c

[34] T. A. Albright, J. K. Burdett, M.-H. Whangbo, Orbital Interactions in Chemistry, John Wiley & Sons, Inc., 2013.

[35] The HOMOs of ClF-MA shift in energy upon interacting with the diene along the reaction coordinate, therefore, the HOMO-3_ClF-MA_ of the equilibrium geometry of Cl-MA in Figure 4 is the same orbital as the HOMO-2_ClF-MA_ of the consistent geometry of Cl-MA in Figure 3.

