# Supplementary material for: Lewis Acid‐Catalyzed Diels‐Alder Reactions: Reactivity Trends across the Periodic Table
Source: Chemistry. 2021 May 1;27(41):10610–20. doi: 10.1002/chem.202100522 (PMC8360170; doi:10.1002/chem.202100522)
Supplement: Supplementary file 1 — Supporting Information [file CHEM-27-10610-s001.pdf]

Chemistry–A European Journal

Supporting Information

Lewis Acid-Catalyzed Diels-Alder Reactions: Reactivity Trends across the Periodic Table

Pascal Vermeeren[+], Marco Dalla Tiezza[+], Michelle van Dongen[+], Israel Fernández,* F. Matthias Bickelhaupt,* and Trevor A. Hamlin*

Contents

Table S1. Energy decomposition analysis terms (in kcal mol^{-1}) and LA•••O=C distance (in Å) computed on LA–methyl acrylate adducts.

Figure S1. Transition state structures for the uncatalyzed and Lewis acid-catalyzed Diels–Alder reaction between **B** and **LA-MA**, computed at ZORA-BP86/TZ2P.

Table S2. Electronic reactant complex energies (ΔE_{RC}), reaction barriers ($\Delta E^{\ddagger}$), and reaction energies (ΔE_{rxn}) (in kcal mol^{-1}) for the uncatalyzed and Lewis acid-catalyzed Diels-Alder reaction between 1,3-butadiene (**B**) and methyl acrylate (**LA-MA**).

Figure S2. a) Activation strain analyses and b) energy decomposition analyses of the uncatalyzed and LA-catalyzed Diels-Alder reaction between **B** and **LA-MA**, where the energy values are plotted from the reactants to the transition state and projected onto the shorter newly forming C_{B}•••C_{β} bond between **B** and **LA-MA**, computed at ZORA-BP86/TZ2P.

Figure S3. a) Activation strain analyses and b) energy decomposition analyses of the uncatalyzed and LA-catalyzed Diels-Alder reaction between **B** and **LA-MA**, where the energy values are plotted from the reactants to the transition state and projected onto the shorter newly forming C_{B}•••C_{β} bond between **B** and **LA-MA**, computed at ZORA-BP86/TZ2P.

Figure S4. a) Activation strain analyses and b) energy decomposition analyses of the uncatalyzed and ClF-catalyzed Diels-Alder reaction between **B** and **LA-MA**, where the energy values are plotted from the reactants to the transition state and projected onto the shorter newly forming C_{B}•••C_{β} bond between **B** and **LA-MA**, computed at ZORA-BP86-D3(BJ)/TZ2P.

Figure S5. a) Activation strain analyses and b) energy decomposition analyses of the asynchronous (black) and constraint synchronous (red) Diels-Alder reactions between **B** and **PF_3-MA**, where the transition states are indicated with a dot, the energy values are plotted from the reactants to the cycloadduct, and projected onto the shorter forming C_{B}•••C_{β} bond between **B** and **PF_3-MA**, computed at ZORA-BP86/TZ2P.

Figure S6. a) Activation strain analyses and b) energy decomposition analyses of the asynchronous (black) and constraint synchronous (red) Diels-Alder reactions between **B** and **SF_2-MA**, where the transition states are indicated with a dot, the energy values are plotted from the reactants to the cycloadduct, and projected onto the shorter forming C_{B}•••C_{β} bond between **B** and **SF_2-MA**, computed at ZORA-BP86/TZ2P.

Figure S7. a) Activation strain analyses and b) energy decomposition analyses of the asynchronous (black) and constraint synchronous (red) Diels-Alder reactions between **B** and **HF-MA**, where the transition states are indicated with a dot, the energy values are plotted from the reactants to the cycloadduct, and projected onto the shorter forming C_{B}•••C_{β} bond between **B** and **HF-MA**, computed at ZORA-BP86/TZ2P.

Figure S8. Key occupied LUMOs (isovalue = 0.03 $Bohr^{-3/2}$) computed at the equilibrium structures of **ClF-MA** and **MA**, where the MO-coefficients of the α-carbon and β-carbon $2p_z$ atomic orbitals, contributing to the occupied orbitals, are shown in the schematic LUMOs, computed at ZORA-BP86/TZ2P.

Figure S9. Molecular orbital diagrams with orbital energies and overlaps for a) normal electron demand (NED) $HOMO_{B}$–**$LUMO_{LA-MA}$** interactions; and for b) inverse electron demand (IED) $LUMO_{B}$–π-MO_{LA-MA} interactions of the asynchronous (black) and constraint synchronous (red) Diels-Alder reactions between **B** and **MA** and **ClF-MA**, computed on consistent geometries with a C_{B}•••$C_{β}$ bond length between **B** and **LA-MA** of 2.128 Å at ZORA-BP86/TZ2P.

Figure S10. a) Activation strain analyses and b) energy decomposition analyses of the **SF_2**-, **SeF_2**-, and **TeF_2**-catalyzed Diels-Alder reaction between **B** and **LA-MA**, where the energy values are plotted from the reactants to the transition state and projected onto the shorter newly forming C_{B}•••$C_{β}$ bond between **B** and **LA-MA**, computed at ZORA-BP86/TZ2P.

Figure S11. a) Activation strain analyses and b) energy decomposition analyses of the **ClF**-, **BrF**-, and **IF**-catalyzed Diels-Alder reaction between **B** and **LA-MA**, where the energy values are plotted from the reactants to the transition state and projected onto the shorter newly forming C_{B}•••$C_{β}$ bond between **B** and **LA-MA**, computed at ZORA-BP86/TZ2P.

Table S3. Cartesian coordinates (in Å), energies (in kcal mol^{-1}), and number of imaginary frequencies of all stationary points, computed at ZORA-BP86/TZ2P.

Table S4. Cartesian coordinates (in Å), energies (in kcal mol^{-1}), and number of imaginary frequencies of all stationary points, computed at ZORA-BP86-D3(BJ)/TZ2P.

Table S1. Energy decomposition analysis terms (in kcal mol^{-1}) and LA•••O=C distance (in Å) computed on LA–methyl acrylate adducts.[a]

LA	ΔE	ΔE_{strain}	ΔE_{int}	ΔV_{elstat}	ΔE_{Pauli}	ΔE_{oi}	ΔE_{disp}	r(LA•••O=C)
PF_3	–4.3	0.3	–4.6	–8.0	10.3	–3.9	–3.0	2.845
AsF_3	–8.5	0.7	–9.3	–16.2	19.3	–8.6	–3.7	2.651
SbF_3	–11.9	1.1	–13.0	–23.2	27.2	–12.5	–4.6	2.654
SF_2	–6.7	0.8	–7.5	–14.9	21.3	–10.9	–3.0	2.515
SeF_2	–10.7	1.3	–11.9	–22.4	29.9	–15.8	–3.7	2.471
TeF_2	–14.1	1.5	–15.6	–28.9	36.8	–19.5	–4.0	2.503
ClF	–11.5	1.0	–12.5	–22.4	34.7	–22.5	–2.4	2.283
BrF	–14.0	1.5	–15.5	–26.0	36.4	–23.1	–2.8	2.349
IF	–15.0	1.3	–16.3	–27.6	35.9	–21.1	–3.5	2.491
HF	–11.5	1.0	–12.5	–18.2	20.6	–13.7	–1.2	1.609

[a] Computed at ZORA-BP86-D3(BJ)/TZ2P.

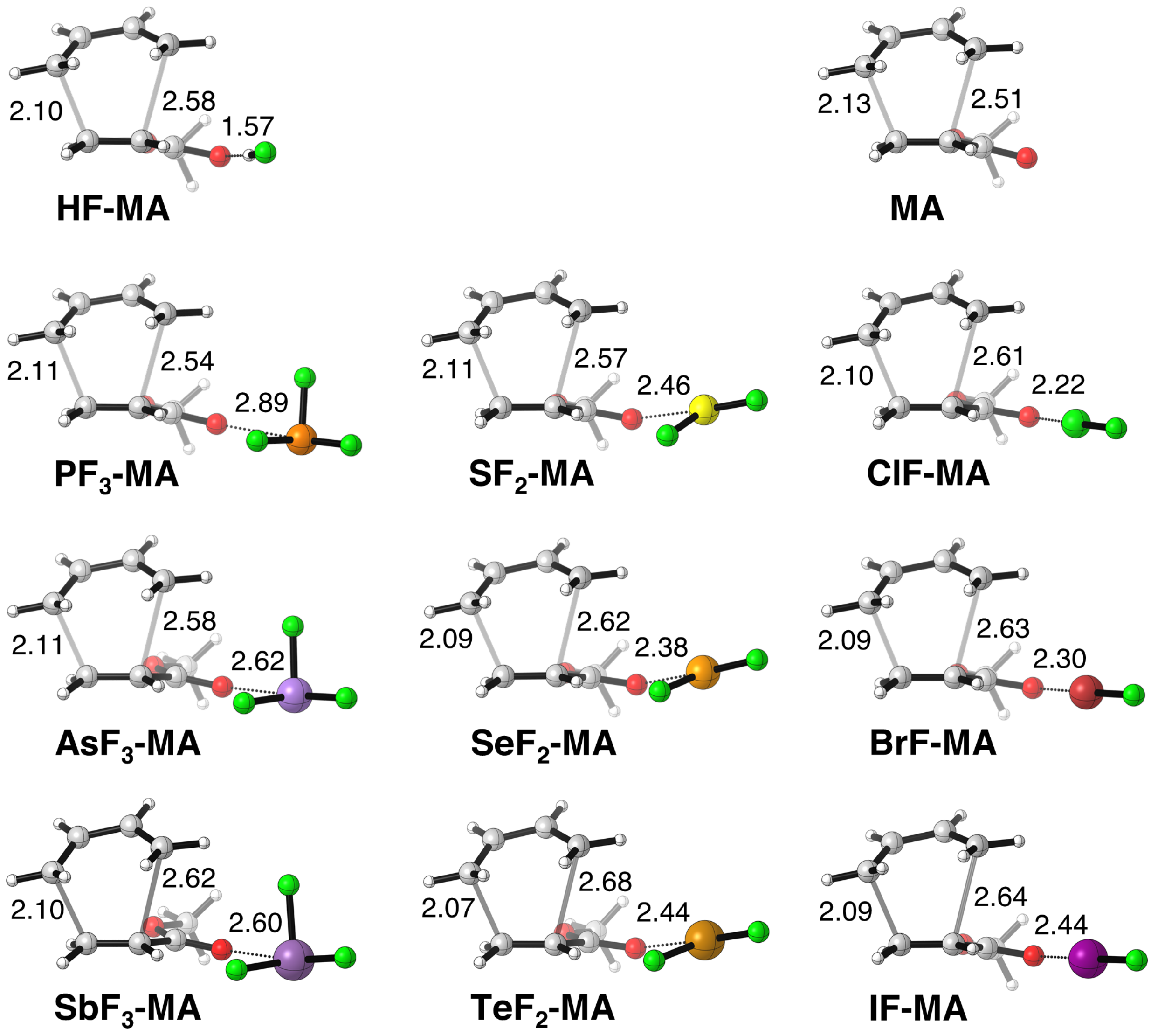

Figure S2. Transition state structures for the uncatalyzed and Lewis acid-catalyzed Diels–Alder reaction between **B** and **LA-MA**, computed at ZORA-BP86/TZ2P.

Table S2. Electronic reactant complex energies (ΔE_{RC}), reaction barriers ($\Delta E^{\ddagger}$), and reaction energies (ΔE_{rxn}) (in kcal mol^{-1}) for the uncatalyzed and Lewis acid-catalyzed Diels-Alder reaction between 1,3-butadiene (**B**) and methyl acrylate (**LA-MA**).[a]

LA-MA	ΔE_{RC}	$\Delta E^{\ddagger}$	ΔE_{rxn}
MA	–3.9	4.4	–44.3
PF_3-MA	–4.8	2.8	–46.3
AsF_3-MA	–5.3	1.4	–47.4
SbF_3-MA	–5.9	0.1	–48.2
SF_2-MA	–5.1	1.9	–44.6
SeF_2-MA	–6.2	0.4	–45.4
TeF_2-MA	–6.6	–0.3	–45.3
ClF-MA	–5.8	0.6	–44.3
BrF-MA	–6.1	0.1	–44.5
IF-MA	–6.5	–0.4	–44.7
HF-MA	–5.0	1.9	–43.8

[a] Computed at ZORA-BP86-D3(BJ)/TZ2P.

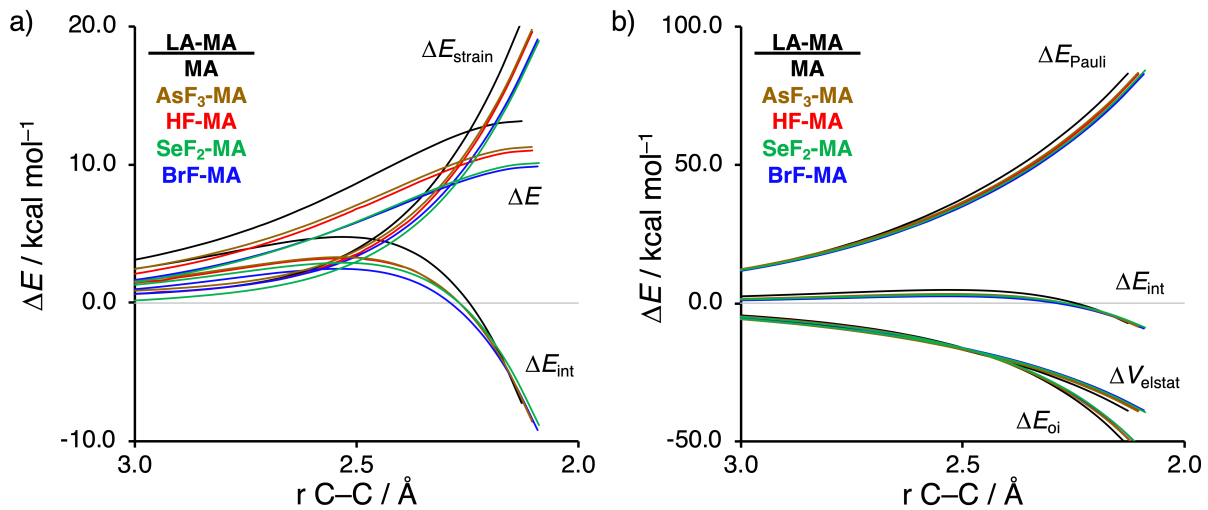

Figure S2. a) Activation strain analyses and b) energy decomposition analyses of the uncatalyzed and LA-catalyzed Diels-Alder reaction between **B** and **LA-MA**, where the energy values are plotted from the reactants to the transition state and projected onto the shorter newly forming C_B•••C_β bond between **B** and **LA-MA**, computed at ZORA-BP86/TZ2P.

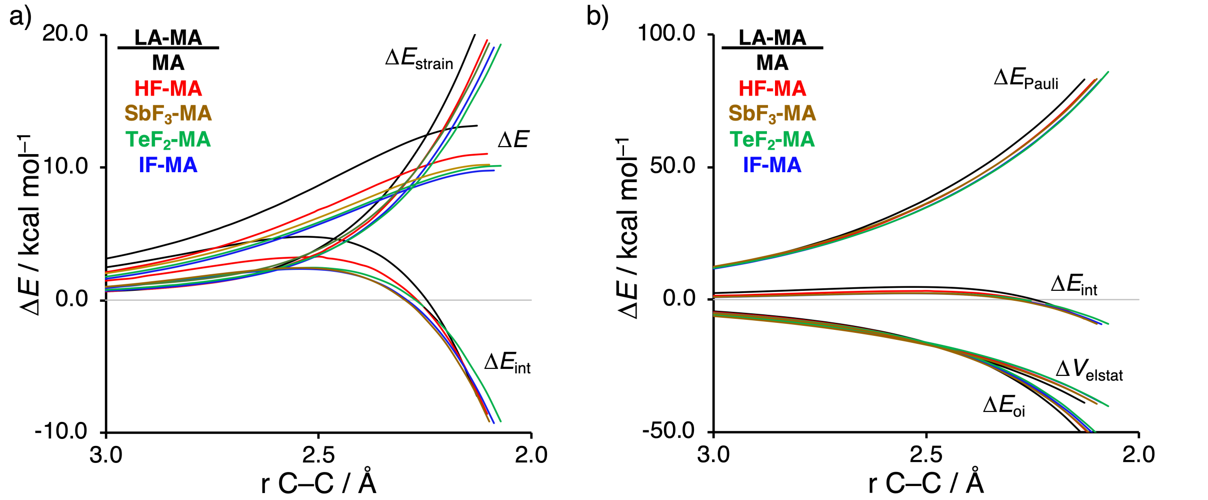

Figure S3. a) Activation strain analyses and b) energy decomposition analyses of the uncatalyzed and LA-catalyzed Diels-Alder reaction between **B** and **LA-MA**, where the energy values are plotted from the reactants to the transition state and projected onto the shorter newly forming C_B•••C_β bond between **B** and **LA-MA**, computed at ZORA-BP86/TZ2P.

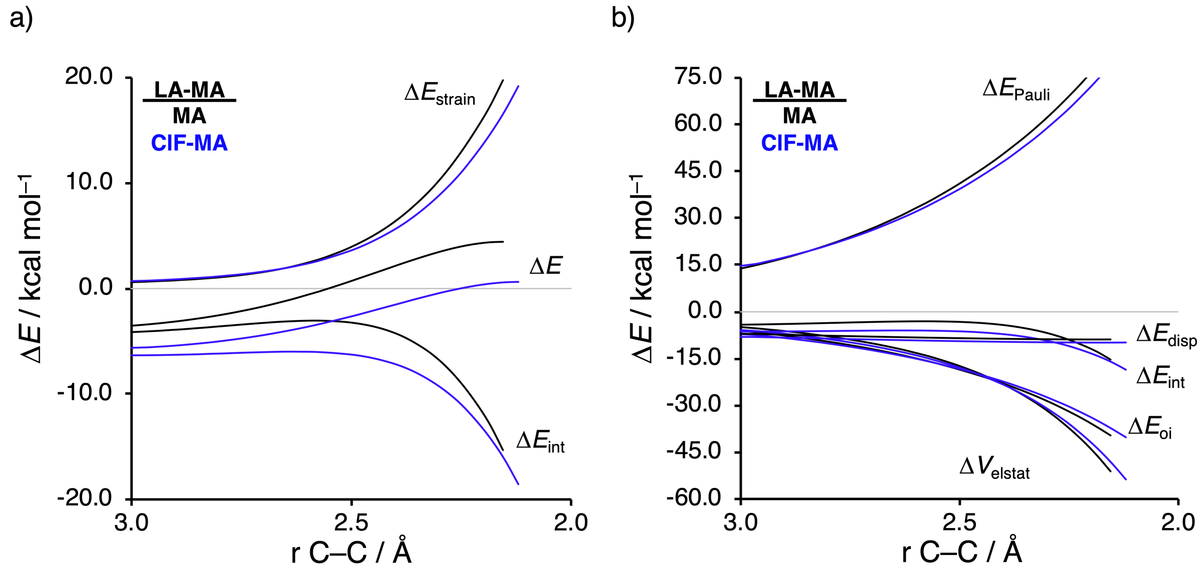

Figure S4. a) Activation strain analyses and b) energy decomposition analyses of the uncatalyzed and ClF-catalyzed Diels-Alder reaction between **B** and **LA-MA**, where the energy values are plotted from the reactants to the transition state and projected onto the shorter newly forming C_B•••C_β bond between **B** and **LA-MA**, computed at ZORA-BP86-D3(BJ)/TZ2P.

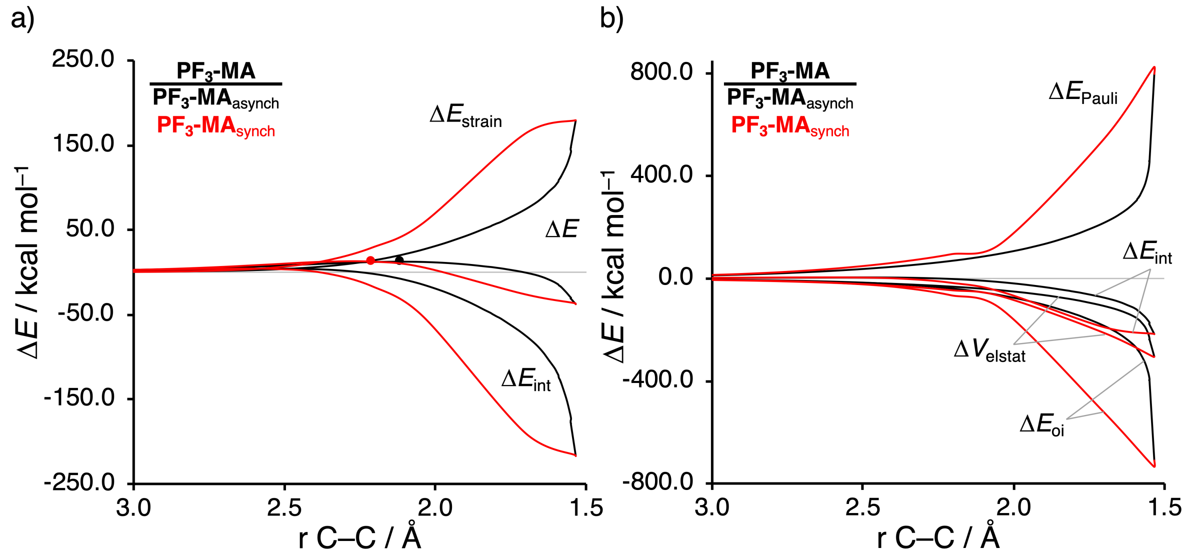

Figure S5. a) Activation strain analyses and b) energy decomposition analyses of the asynchronous (black) and constraint synchronous (red) Diels-Alder reactions between **B** and **PF_3-MA**, where the transition states are indicated with a dot, the energy values are plotted from the reactants to the cycloadduct, and projected onto the forming C_B•••C_β bond between **B** and **PF_3-MA**, computed at ZORA-BP86/TZ2P.

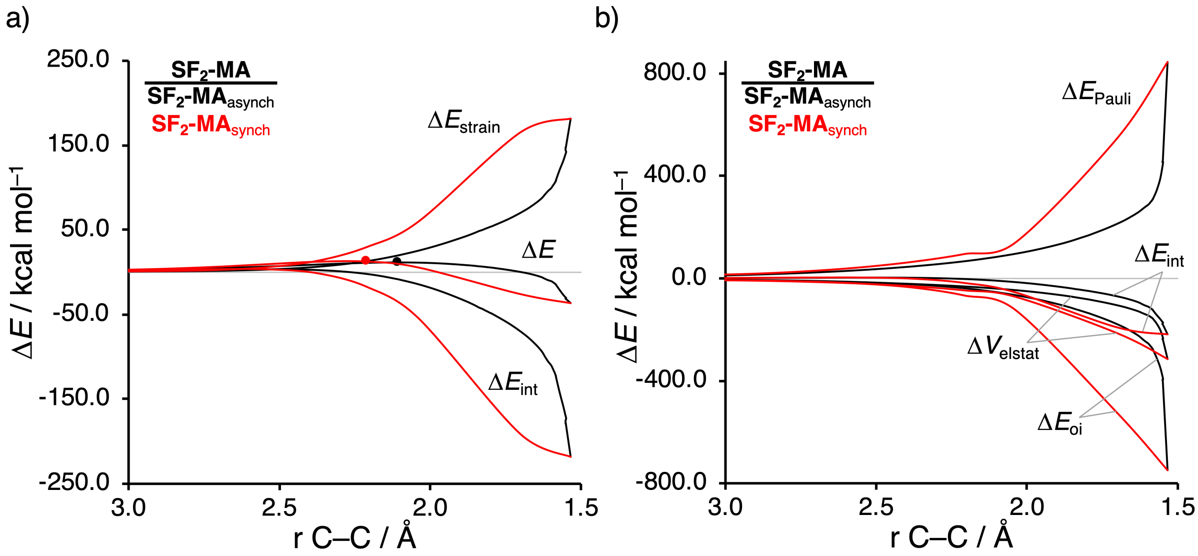

Figure S6. a) Activation strain analyses and b) energy decomposition analyses of the asynchronous (black) and constraint synchronous (red) Diels-Alder reactions between **B** and **SF_2-MA**, where the transition states are indicated with a dot, the energy values are plotted from the reactants to the cycloadduct, and projected onto the shorter forming $C_B\cdots C_\beta$ bond between **B** and **SF_2-MA**, computed at ZORA-BP86/TZ2P.

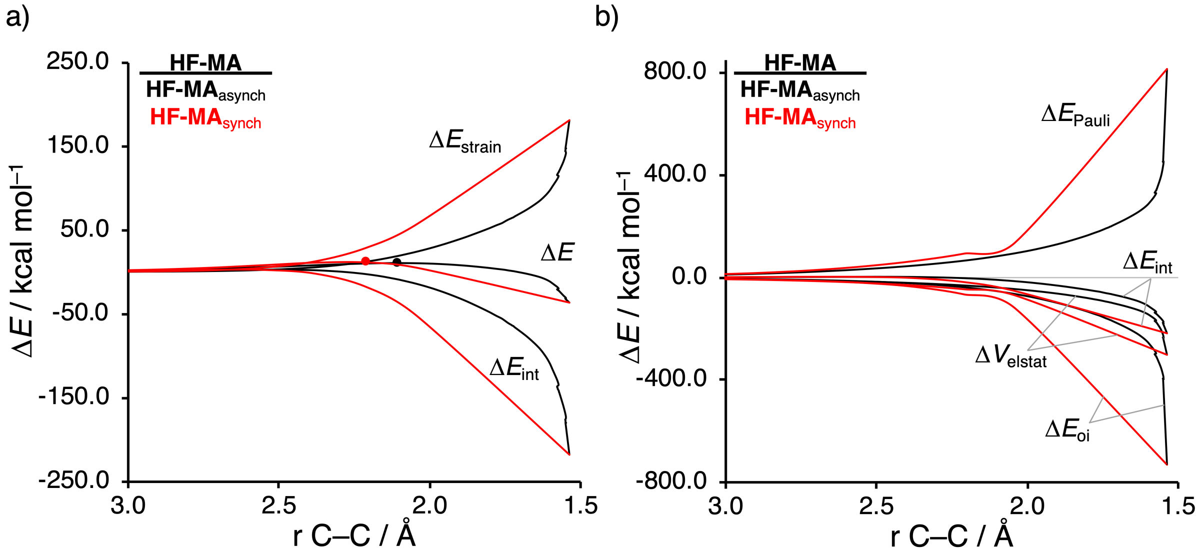

Figure S7. a) Activation strain analyses and b) energy decomposition analyses of the asynchronous (black) and constraint synchronous (red) Diels-Alder reactions between **B** and **HF-MA**, where the transition states are indicated with a dot, the energy values are plotted from the reactants to the cycloadduct, and projected onto the shorter forming $C_B\cdots C_\beta$ bond between **B** and **HF-MA**, computed at ZORA-BP86/TZ2P.

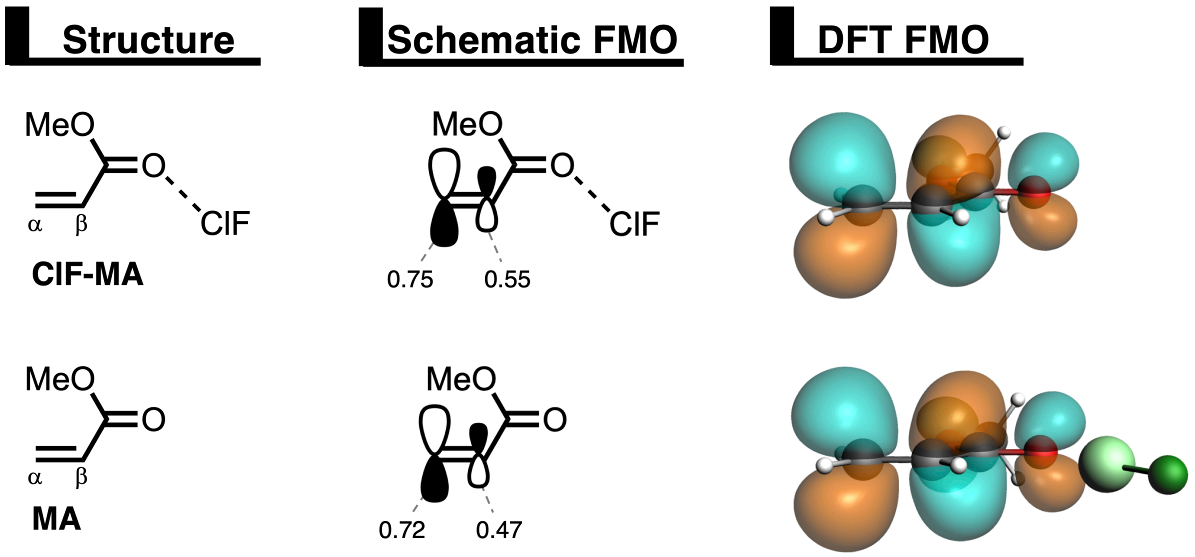

Figure S8. Key occupied LUMOs (isovalue = 0.03 Bohr$^{-3/2}$) computed at the equilibrium structures of **ClF-MA** and **MA**, where the MO-coefficients of the α-carbon and β-carbon $2p_z$ atomic orbitals, contributing to the occupied orbitals, are shown in the schematic LUMOs, computed at ZORA-BP86/TZ2P.

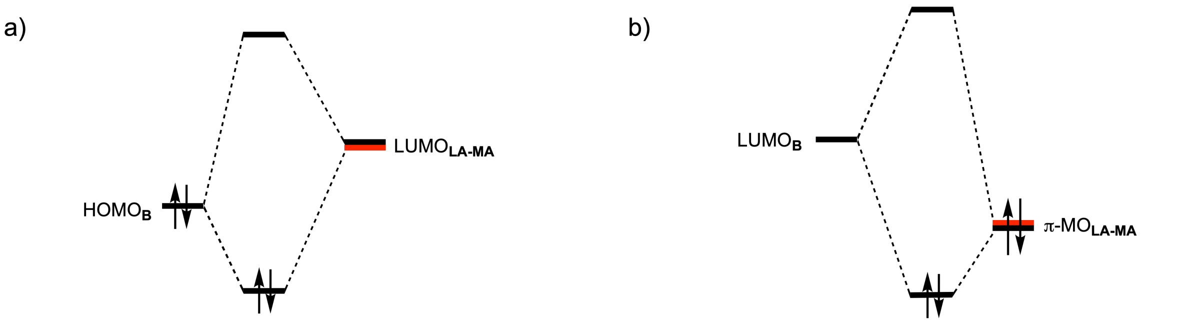

LA-MA	Δε HOMO$_B$–LUMO$_{LA\text{-}MA}$ (eV)	Orbital Overlap *S*
**MA**$_{asynch}$	2.5	0.20
**MA**$_{synch}$	2.1	0.24
**ClF-MA**$_{asynch}$	1.8	0.19
**ClF-MA**$_{synch}$	1.4	0.23

LA-MA	Δε LUMO$_B$–π-MO$_{LA\text{-}MA}$ (eV)	Orbital Overlap *S*
**MA**$_{asynch}$	HOMO–1 4.4	0.18
**MA**$_{synch}$	HOMO–1 4.0	0.21
**ClF-MA**$_{asynch}$	HOMO–2 5.2	0.15
**ClF-MA**$_{synch}$	HOMO–2 4.6	0.21

Figure S9. Molecular orbital diagrams with orbital energies) and overlaps for a) normal electron demand (NED) HOMO$_B$–**LUMO**$_{LA\text{-}MA}$ interactions; and for b) inverse electron demand (IED) LUMO$_B$–π-MO$_{LA\text{-}MA}$ interactions of the asynchronous (black) and constraint synchronous (red) Diels-Alder reactions between **B** and **MA** and **ClF-MA**, computed on consistent geometries with a $C_B \cdots C_\beta$ bond length between **B** and **LA-MA** of 2.128 Å at ZORA-BP86/TZ2P.

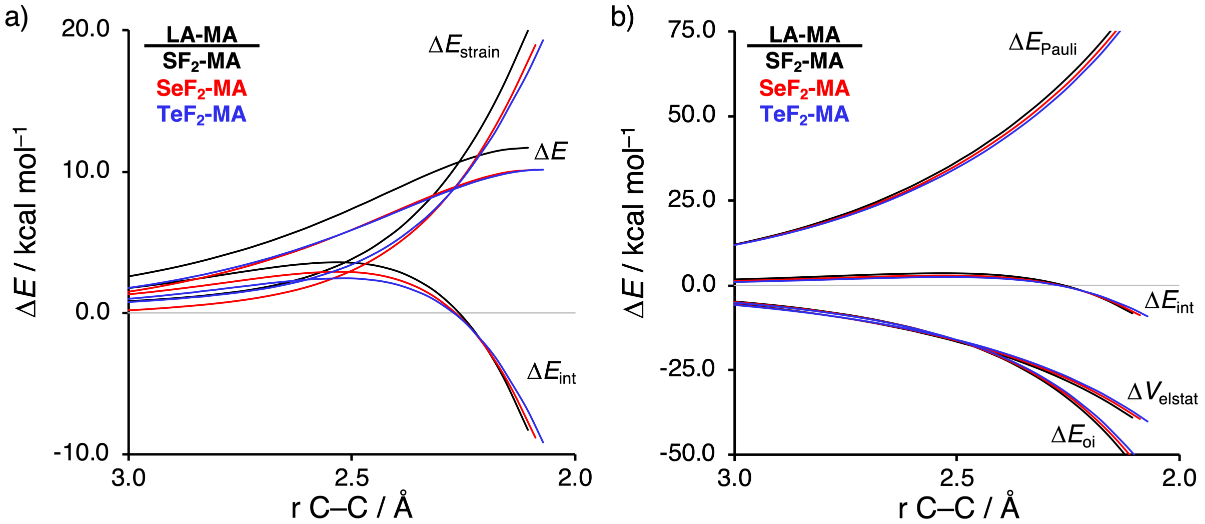

Figure S10. a) Activation strain analyses and b) energy decomposition analyses of the $\mathbf{SF_2}$-, $\mathbf{SeF_2}$-, and $\mathbf{TeF_2}$-catalyzed Diels-Alder reaction between **B** and **LA-MA**, where the energy values are plotted from the reactants to the transition state and projected onto the shorter newly forming C_B•••C_β bond between **B** and **LA-MA**, computed at ZORA-BP86/TZ2P.

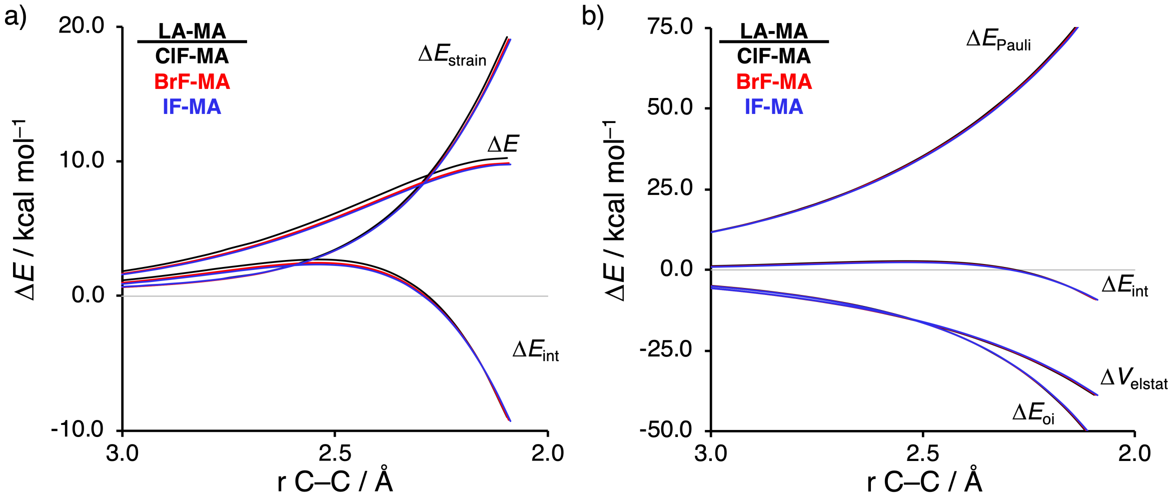

Figure S11. a) Activation strain analyses and b) energy decomposition analyses of the **ClF**-, **BrF**-, and **IF**-catalyzed Diels-Alder reaction between **B** and **LA-MA**, where the energy values are plotted from the reactants to the transition state and projected onto the shorter newly forming C_B•••C_β bond between **B** and **LA-MA**, computed at ZORA-BP86/TZ2P.

Table S3. Cartesian coordinates (in Å), energies (in kcal mol^{-1}), and number of imaginary frequencies of all stationary points, computed at ZORA-BP86/TZ2P.

1,3-butadiene (B)
E = -1293.94
H = -1238.68
G = -1259.06
$\mathbf{N_{imag}}$ = 0

C	0.285526	0.583234	-0.872912
C	0.501489	-0.197985	0.194956
C	-0.531284	-0.805011	1.039169
C	-1.744789	-0.292651	1.288479
H	-0.722451	0.807668	-1.223603
H	1.113333	1.008062	-1.438519
H	1.534334	-0.438969	0.463251
H	-0.255613	-1.748075	1.520271
H	-2.461279	-0.818210	1.917837
H	-2.052225	0.673131	0.885844

HF
E = −181.82
H = −174.09
G = − 186.47
$\mathbf{N_{imag}}$ = 0

F	-1.097649	0.981173	0.000000
H	-0.165896	0.923022	0.000000

ClF
E = -93.36
H = -90.14
G = -105.68
$\mathbf{N_{imag}}$ = 0

Cl	-2.261081	-0.407459	-0.000001
F	-0.602470	-0.288958	-0.000000

BrF
E = -91.79
H = -88.71
G = -105.04
$\mathbf{N_{imag}}$ = 0

Br	-1.213880	-0.151645	0.000062
F	-2.985688	0.140194	-0.000121

IF
E = −96.04
H = −93.03
G = −109.87
$\mathbf{N_{imag}}$ = 0

I	-1.140017	-0.163811	0.000070
F	-3.059551	0.152360	-0.000129

SF_2
E = −243.74
H = −238.33

G = -256.8
$\mathbf{N_{imag}}$ = 0

S	0.160283	-0.118898	0.000000
F	-1.058702	-1.193509	0.000000
F	1.422609	-1.142195	0.000000

SeF_2
E = -231.8
H = -226.76
G = -246.23
$\mathbf{N_{imag}}$ = 0

Se	-0.560331	-0.335823	0.004079
F	0.266590	0.463222	-1.340533
F	0.267190	0.492708	1.330142

TeF_2
E = -234.66
H = -229.74
G = -249.90
$\mathbf{N_{imag}}$ = 0

Te	-0.623674	-0.398139	0.004686
F	0.298208	0.493317	-1.434204
F	0.298915	0.524929	1.423206

PF_3
E = -461.14
H = -452.95
G = -473.25
$\mathbf{N_{imag}}$ = 0

P	-0.490029	-0.184345	-0.022635
F	-1.754324	-1.093865	-0.390295
F	0.645797	-1.144830	-0.613106
F	-0.583039	0.825869	-1.260283

AsF_3
E = -420.59
H = -413.17
G = -433.63
$\mathbf{N_{imag}}$ = 0

As	0.019840	0.010305	-0.664527
F	1.503865	0.010202	0.265972
F	-0.749084	-1.318215	0.179850
F	-0.774621	1.297708	0.218705

SbF_3
E = -413.32
H = -406.15
G = -427.95
$\mathbf{N_{imag}}$ = 0

Sb	0.021638	0.011246	-0.749729
F	1.631635	0.011464	0.298456
F	-0.813129	-1.431961	0.204398
F	-0.840143	1.409251	0.246875

Methyl acrylate (MA)
E = –1618.47

H = -1555.90
G = -1580.08
$\mathbf{N_{imag}}$ **=** 0

O	1.273582	0.800853	-0.616230
C	2.122368	1.847847	-0.096294
H	-0.568804	-0.274204	-1.915548
H	-0.931445	-2.102607	-1.944528
H	0.955099	-2.502956	-0.403778
H	3.163962	1.680664	-0.394980
H	2.066971	1.878018	0.998296
H	1.738877	2.775125	-0.529531
O	2.517248	-0.690022	0.569762
C	-0.319502	-1.279029	-1.578306
C	0.700772	-1.499222	-0.744614
C	1.593441	-0.453186	-0.189177

HF-MA
E = -1810.55
H = -1738.71
G = -1766.72
$\mathbf{N_{imag}}$ = 0

C	3.713227	2.476867	0.000095
C	2.714326	1.589061	0.000135
C	2.901511	0.124094	-0.000059
O	1.968694	-0.681362	-0.000027
O	4.187365	-0.271592	-0.000279
C	4.398156	-1.705909	-0.000482
H	4.755696	2.162488	-0.000101
H	3.507241	3.546275	0.000251
H	1.667801	1.895457	0.000329
H	3.949910	-2.154303	-0.894013
H	3.950153	-2.154520	0.893062
H	5.482720	-1.835240	-0.000645
H	0.461081	-0.093267	0.000219
F	-0.398062	0.349332	0.000367

ClF-MA
E = -1720.35
H = -1653.52
G = -1683.87
$\mathbf{N_{imag}}$ = 0

C	3.916881	2.462880	0.000407
C	2.917362	1.575549	0.000516
C	3.109010	0.114550	-0.000102
O	2.188115	-0.710038	-0.000002
O	4.394283	-0.278902	-0.000797
C	4.613708	-1.713300	-0.001409
H	4.959583	2.149270	-0.000168
H	3.710271	3.532127	0.000892
H	1.871673	1.880639	0.001081
H	4.167956	-2.163237	-0.895231
H	4.168782	-2.163872	0.892505
H	5.699106	-1.834312	-0.001953
Cl	-0.017872	-0.086713	0.001038
F	-1.687348	0.335882	0.001806

BrF-MA
E = -1721.45
H = -1654.73
G = -1685.77
$\mathbf{N_{imag}}$ = 0

C	3.972518	2.459799	-0.010964
C	2.963783	1.582858	-0.005906
C	3.140652	0.121276	0.000471
O	2.209924	-0.695275	0.005302
O	4.418595	-0.288312	0.000781
C	4.623796	-1.725478	0.006940
H	5.011997	2.135578	-0.010675
H	3.776678	3.531019	-0.015514
H	1.920710	1.898622	-0.006156
H	4.173102	-2.174754	-0.884625
H	4.175391	-2.166760	0.903636
H	5.707937	-1.856508	0.006143
Br	-0.069318	-0.108038	0.004825
F	-1.874629	0.303589	0.004607

IF-MA
E = -1726.09
H = -1659.45
G = -1691.16
$\mathbf{N_{imag}}$ = 0

C	4.033459	2.470631	-0.012975
C	3.016484	1.603226	-0.003877
C	3.177430	0.139783	0.001898
O	2.236568	-0.665039	0.008740
O	4.450509	-0.283461	-0.000694
C	4.639338	-1.722922	0.004211
H	5.069700	2.136138	-0.016714
H	3.848312	3.543758	-0.017006
H	1.976488	1.931157	-0.000381
H	4.181001	-2.166426	-0.886322
H	4.188208	-2.159375	0.901879
H	5.721857	-1.866450	0.000436
I	-0.216035	-0.153737	0.005578
F	-2.172199	0.210281	0.001797

SF_2-MA
E = -1865.96
H = -1797.33
G = -1829.72
$\mathbf{N_{imag}}$ = 0

S	-3.294457	0.396900	-0.242360
F	-2.641407	0.443220	1.257749
F	-4.760476	-0.211888	0.203691
O	1.190095	1.539199	-1.272290
C	0.995988	2.903156	-1.718826
H	2.410358	-0.527682	-0.593040
H	1.600010	-2.097745	0.000067
H	-0.620743	-1.040534	-0.219497
H	0.516066	3.495861	-0.932026
H	0.368950	2.923174	-2.617372
H	1.998398	3.279710	-1.935216
O	-1.048794	1.402035	-0.999980
C	1.498904	-1.070809	-0.348436
C	0.294977	-0.503389	-0.466288
C	0.061337	0.881284	-0.927287

SeF2-MA
E = -1857.31
H = -1789.00
G = -1821.68
$\mathbf{N_{imag}}$ = 0

Se	-3.181786	0.357458	-0.371408
F	-2.614695	0.234721	1.318190
F	-4.696544	-0.572402	-0.076718
O	1.180208	1.575270	-1.226213
C	1.021374	2.922689	-1.736986
H	2.266966	-0.661433	-0.913891
H	1.469098	-2.104656	-0.044994
H	-0.616140	-0.817597	0.206357
H	0.666988	3.588124	-0.942039
H	0.305414	2.933922	-2.566130
H	2.016959	3.217099	-2.076544
O	-1.031595	1.561425	-0.787535
C	1.394311	-1.084741	-0.419464
C	0.260982	-0.391043	-0.277862
C	0.059170	0.983553	-0.775297

TeF2-MA
E = -1863.27
H = -1794.47
G = -1829.06
$\mathbf{N_{imag}}$ = 0

C	1.757942	1.522783	1.811279
C	0.632890	1.097357	1.225717
C	0.303585	1.326199	-0.189536
O	-0.709139	0.881342	-0.746368
O	1.201869	2.068209	-0.861946
C	0.912915	2.298282	-2.262988
Te	-2.611660	-0.707741	-0.303532
F	-4.021901	-1.979549	0.171863
F	-2.060271	-0.580133	1.560160

H	2.512856	2.083677	1.262684
H	1.948881	1.312545	2.862553
H	-0.125074	0.530014	1.764516
H	-0.026266	2.852571	-2.369798
H	0.836227	1.344690	-2.796984
H	1.755185	2.885886	-2.634838

PF_3-MA
E = -2081.21
H = -2010.41
G = -2043.36
$\mathbf{N_{imag}}$ = 0

F	-2.250945	0.302351	1.712290
H	0.140499	-3.209136	-1.916184
H	0.629186	-3.246655	-0.199419
F	-4.382730	1.155782	1.002804
H	1.835701	-3.636377	-1.479746
H	2.975430	0.255399	-1.693888
H	2.517603	2.054680	-1.528011
H	0.215692	1.416364	-0.909610
C	2.221236	1.008070	-1.470090
C	0.973740	0.666920	-1.134888
C	0.474388	-0.723188	-1.027750
O	-0.672033	-1.009201	-0.712241
O	1.416680	-1.656799	-1.316931
C	0.966660	-3.027341	-1.219025
P	-3.115858	0.391126	0.366286
F	-2.406873	1.694408	-0.254211

AsF_3-MA
E = -2044.03
H = -1973.35
G = -2008.29
$\mathbf{N_{imag}}$ = 0

F	-1.718966	0.701657	1.628901
H	0.110747	-3.210135	-1.966288
H	0.588255	-3.291021	-0.246156
F	-4.226492	1.142322	1.099724
H	1.811338	-3.613470	-1.529372
H	2.890452	0.289520	-1.562910
H	2.398277	2.075985	-1.363810
H	0.089826	1.387846	-0.827199
C	2.117640	1.023853	-1.341685
C	0.866154	0.657107	-1.049718
C	0.396979	-0.742916	-0.991603
O	-0.748316	-1.069630	-0.688510
O	1.347516	-1.649987	-1.308262
C	0.926683	-3.033528	-1.256189
As	-2.873897	0.257578	0.377547
F	-2.441885	1.549444	-0.744363

SbF_3-MA
E = -2039.25
H = -1968.77
G = -2004.00
$\mathbf{N_{imag}}$ = 0

F	-1.627032	0.365411	1.706257
H	0.157102	-3.233256	-1.951681
H	0.539985	-3.250304	-0.205087
F	-4.234002	1.265833	1.215374
H	1.834568	-3.602653	-1.407616
H	2.884170	0.306092	-1.501934
H	2.367500	2.092670	-1.384011
H	0.032534	1.399074	-0.968692
C	2.093761	1.039151	-1.349073
C	0.828640	0.671510	-1.122894
C	0.372198	-0.729711	-1.056352
O	-0.792518	-1.063921	-0.824863
O	1.341312	-1.638131	-1.279233
C	0.930353	-3.024729	-1.203815
Sb	-2.919281	0.179748	0.284707
F	-2.269944	1.695491	-0.732752

TS: B + MA
E = -2899.25
H = -2780.56
G = -2811.38
$\mathbf{N_{imag}}$ = 1, ν = -402i cm^{-1}

C	-0.092633	-0.263357	1.633832
C	-1.441080	0.014565	1.326674
C	-2.209044	-0.834339	0.543058
C	0.545826	-1.400761	1.205601
H	-1.812305	1.020427	1.530583
H	-3.224617	-0.542055	0.277151
H	-2.048618	-1.909614	0.597963
H	-0.004465	-2.304341	0.958420
H	1.621899	-1.517388	1.320545
C	-1.243318	-0.830411	-1.352780
C	0.105612	-1.158031	-1.248453
C	1.196651	-0.182965	-1.377629
O	2.369140	-0.463466	-1.577308
O	0.750495	1.112819	-1.278153
C	1.782577	2.109701	-1.413089
H	-1.509917	0.194713	-1.595863
H	-1.929543	-1.594060	-1.716659
H	0.435960	-2.187550	-1.370312
H	2.268686	2.032700	-2.393182
H	2.544451	1.988129	-0.633265
H	1.275150	3.072885	-1.309209
H	0.503233	0.538777	2.072738

P: B + MA
E = -2949.45
H = -2827.83
G = -2857.51
$\mathbf{N_{imag}}$ = 0

C	2.692349	-1.977888	-0.358933
C	3.942767	-1.560577	-0.133508
C	4.273732	-0.386127	0.748425
C	1.466304	-1.308500	0.203792
H	4.776857	-2.083453	-0.606708

H	4.598009	0.467095	0.126456
H	5.141517	-0.628112	1.381468
H	1.004122	-1.960676	0.964000
H	0.718617	-1.188304	-0.592705
C	3.087288	0.019885	1.631764
C	1.769638	0.062689	0.828914
C	1.746904	1.167952	-0.228106
O	1.348668	1.058728	-1.369906
O	2.191468	2.348886	0.291451
C	2.144178	3.468800	-0.621763
H	3.273465	0.986340	2.114182
H	2.962242	-0.726471	2.430513
H	0.956096	0.330467	1.523580
H	1.113227	3.657127	-0.943971
H	2.761373	3.269597	-1.505558
H	2.535749	4.320174	-0.059009
H	2.524133	-2.847710	-0.997600

TS: B + HF-MA
E = -3093.47
H = -2965.51
G = -3000.17
$\mathbf{N_{imag}}$ = 1, ν = -384i cm^{-1}

C	2.948934	-1.337023	-1.337774
C	3.944251	-1.609671	-0.373199
C	3.663980	-2.256172	0.822399
C	1.640388	-1.714358	-1.184783
H	3.208109	-0.660945	-2.154588
H	4.463798	-2.397196	1.548863
H	2.889011	-3.020301	0.849937
H	1.347726	-2.519695	-0.517217
H	0.866468	-1.362960	-1.864560
C	2.391898	-0.938756	1.858031
C	1.259243	-0.616928	1.114724
C	1.072527	0.664368	0.449409
O	0.000037	1.075478	-0.017308
O	2.192533	1.428963	0.405822
C	2.037133	2.715145	-0.235400
H	3.139884	-0.168054	2.025432
H	2.279482	-1.663835	2.662897
H	0.348333	-1.207198	1.193639
H	1.294142	3.320626	0.296074
H	1.716664	2.590129	-1.276206
H	3.024037	3.182265	-0.186838
H	-1.188521	0.048098	-0.036180
F	-1.864447	-0.655401	-0.044675
H	4.910619	-1.115248	-0.487348

P: B + HF-MA
E = -3140.67
H = -3009.77
G = -3043.28
$\mathbf{N_{imag}}$ = 0

C	-3.141151	-1.964179	0.315240
C	-4.395124	-1.505175	0.233802

C	-4.824021	-0.418346	-0.713844
C	-1.997598	-1.430838	-0.505881
H	-5.164294	-1.930509	0.882432
H	-5.106876	0.479873	-0.140932
H	-5.741505	-0.729438	-1.237771
H	-1.753452	-2.141313	-1.313017
H	-1.086034	-1.362385	0.101811
C	-3.738968	-0.075194	-1.746425
C	-2.324043	-0.060425	-1.140381
C	-2.047096	1.077061	-0.165347
O	-1.035778	1.180019	0.523305
O	-2.998929	2.026464	-0.153620
C	-2.739895	3.162846	0.712223
H	-3.964114	0.879782	-2.236664
H	-3.736685	-0.845092	-2.532102
H	-1.592759	0.117188	-1.947306
H	-1.813657	3.663957	0.411028
H	-2.650399	2.831485	1.752328
H	-3.601623	3.821745	0.583914
H	0.329815	0.322197	0.402343
F	1.141919	-0.189107	0.304517
H	-2.908135	-2.772711	1.011155

TS: B + ClF-MA

E = -3004.04

H = -2881.03

G = -2917.57

$\mathbf{N_{imag}}$ = 1, ν = -376i cm^{-1}

C	2.967865	-1.334713	-1.337760
C	3.962336	-1.596075	-0.367920
C	3.688594	-2.249552	0.825437
C	1.664845	-1.731839	-1.197622
H	3.224533	-0.657014	-2.153995
H	4.491548	-2.387457	1.548966
H	2.920756	-3.020981	0.850295
H	1.375771	-2.533221	-0.523578
H	0.891464	-1.390078	-1.882941
C	2.422983	-0.945653	1.870381
C	1.275664	-0.638843	1.143213
C	1.078208	0.627849	0.467971
O	0.006076	1.046152	-0.012719
O	2.184092	1.408635	0.417216
C	2.018079	2.689148	-0.236437
H	3.163987	-0.166227	2.028322
H	2.327461	-1.669189	2.678840
H	0.375125	-1.241790	1.228970
H	1.269277	3.292111	0.289249
H	1.701581	2.551317	-1.276620
H	3.001392	3.163464	-0.188774
Cl	-1.782469	-0.273202	-0.070204
F	-3.201972	-1.280876	-0.157698
H	4.923720	-1.091382	-0.479305

P: B + ClF-MA

E = -3050.48

H = -2924.5
G = -2959.85
$\mathbf{N_{imag}}$ = 0

C	-3.237041	-1.946249	0.267661
C	-4.485485	-1.575060	-0.035614
C	-4.817606	-0.495475	-1.026680
C	-2.005217	-1.325236	-0.332399
H	-5.325052	-2.070078	0.457605
H	-5.277907	0.353679	-0.497728
H	-5.591099	-0.861575	-1.720227
H	-1.570372	-2.005067	-1.083507
H	-1.223195	-1.201462	0.428874
C	-3.595427	-0.028785	-1.834895
C	-2.290832	0.036656	-1.020792
C	-2.194357	1.161584	-0.009958
O	-1.156345	1.491685	0.571390
O	-3.339852	1.817535	0.221273
C	-3.264734	2.909965	1.177765
H	-3.799928	0.938678	-2.311756
H	-3.413354	-0.746885	-2.648043
H	-1.449022	0.236049	-1.701584
H	-2.539706	3.657702	0.839499
H	-2.964022	2.528828	2.159373
H	-4.273594	3.326904	1.211622
Cl	0.893153	0.736918	-0.084911
F	2.467228	0.229342	-0.577690
H	-3.074888	-2.752163	0.986532

TS: B + BrF-MA
E = -3005.52
H = -2882.63
G = -2919.86
$\mathbf{N_{imag}}$ = 1, ν = -372i cm^{-1}

C	2.967929	-1.339614	-1.338872
C	3.966401	-1.593537	-0.370683
C	3.700469	-2.246455	0.824868
C	1.667975	-1.744644	-1.196814
H	3.219948	-0.664240	-2.158483
H	4.508088	-2.381830	1.543638
H	2.935659	-3.020782	0.853734
H	1.382847	-2.541599	-0.515828
H	0.891749	-1.410942	-1.882984
C	2.443620	-0.946104	1.876323
C	1.288228	-0.644218	1.159720
C	1.081705	0.616788	0.480784
O	0.003839	1.028493	0.001591
O	2.179796	1.405597	0.422428
C	2.003853	2.683240	-0.234962
H	3.182458	-0.163063	2.026839
H	2.357479	-1.667730	2.687558
H	0.391499	-1.253045	1.250682
H	1.255432	3.284402	0.293236
H	1.682470	2.540169	-1.272858
H	2.985213	3.162065	-0.194143
Br	-1.874306	-0.286908	-0.093412

F	-3.421881	-1.327210	-0.218468
H	4.925330	-1.085267	-0.486824

P: B + BrF-MA
E = -3051.61
H = -2925.75
G = -2961.76
$\mathbf{N_{imag}}$ = 0

C	-3.275505	-1.944347	0.230631
C	-4.515118	-1.565560	-0.098078
C	-4.820481	-0.475549	-1.086127
C	-2.027997	-1.323788	-0.335894
H	-5.367633	-2.061911	0.370879
H	-5.292453	0.368936	-0.560085
H	-5.577295	-0.833130	-1.802107
H	-1.579452	-1.997439	-1.084374
H	-1.260977	-1.213048	0.442995
C	-3.578493	-0.003261	-1.860319
C	-2.290893	0.046511	-1.018313
C	-2.208396	1.160232	0.003551
O	-1.170282	1.504890	0.580908
O	-3.361209	1.790287	0.256196
C	-3.300719	2.870303	1.228704
H	-3.768397	0.971052	-2.329375
H	-3.380673	-0.711254	-2.678528
H	-1.432691	0.246032	-1.678730
H	-2.599111	3.640755	0.892245
H	-2.978484	2.481799	2.200431
H	-4.319136	3.261071	1.280833
Br	0.970882	0.764693	-0.016848
F	2.689470	0.243949	-0.481124
H	-3.132647	-2.757204	0.945699

TS: B + IF-MA
E = -3010.26
H = -2887.44
G = -2925.31
$\mathbf{N_{imag}}$ = 1, ν = -372i cm^{-1}

C	2.988524	-1.340069	-1.337630
C	3.989941	-1.585132	-0.370077
C	3.730226	-2.237916	0.827078
C	1.691821	-1.754391	-1.194253
H	3.235471	-0.665937	-2.159783
H	4.540633	-2.368273	1.543638
H	2.970875	-3.017567	0.857759
H	1.412039	-2.549396	-0.508742
H	0.913453	-1.429963	-1.882601
C	2.472940	-0.945533	1.880751
C	1.313815	-0.645979	1.168865
C	1.100628	0.613834	0.490346
O	0.017327	1.022785	0.021326
O	2.198158	1.402277	0.420757
C	2.015380	2.679413	-0.235641
H	3.208575	-0.159210	2.030155
H	2.391341	-1.667218	2.692464

H	0.419537	-1.259010	1.263623
H	1.269772	3.279489	0.297698
H	1.686707	2.535481	-1.271125
H	2.996179	3.159921	-0.202206
I	-2.047835	-0.276426	-0.112143
F	-3.754888	-1.313104	-0.272297
H	4.945445	-1.071081	-0.488821

P: B + IF-MA
E = -3056.29
H = -2930.50
G = -2967.17
$\mathbf{N_{imag}}$ = 0

C	-3.307167	-1.947088	0.231719
C	-4.545110	-1.563545	-0.097729
C	-4.845983	-0.474707	-1.088371
C	-2.057388	-1.333476	-0.337512
H	-5.399712	-2.054951	0.372616
H	-5.314961	0.372657	-0.564330
H	-5.603887	-0.831080	-1.803776
H	-1.613327	-2.010067	-1.086018
H	-1.288227	-1.226760	0.440418
C	-3.602068	-0.008732	-1.863323
C	-2.314648	0.037333	-1.020864
C	-2.226783	1.152016	-0.000408
O	-1.183924	1.496536	0.568403
O	-3.378047	1.781132	0.259313
C	-3.311302	2.861652	1.230929
H	-3.787851	0.965692	-2.333771
H	-3.406984	-0.718719	-2.680448
H	-1.455263	0.232533	-1.682094
H	-2.613304	3.632794	0.888628
H	-2.980824	2.473829	2.200134
H	-4.329704	3.251237	1.290954
I	1.144222	0.809729	-0.003925
F	3.022239	0.325754	-0.459646
H	-3.167607	-2.758859	0.948621

TS: B + SF_2-MA
E = -3148.24
H = -3022.86
G = -3062.87
$\mathbf{N_{imag}}$ = 1, ν = -387i cm^{-1}

C	2.560474	-1.274917	-0.553969
C	3.619593	-0.673557	0.160893
C	3.529288	-0.379857	1.514334
C	1.379149	-1.643327	0.035838
H	4.359333	0.129605	2.003068
H	2.954056	-1.033859	2.167454
H	1.304187	-1.822435	1.104739
H	0.523812	-1.962957	-0.557053
C	2.011086	1.073022	1.651087
C	0.820486	0.643004	1.070328
C	0.349846	1.104402	-0.228993
O	-0.787020	0.939897	-0.686424

O	1.291333	1.799896	-0.926467
C	0.864529	2.288312	-2.216601
S	-2.573668	-0.670561	-0.178274
F	-3.780982	-1.786569	0.028370
F	-2.277576	-0.455862	1.424125
H	2.574236	1.860983	1.158021
H	2.077011	1.060770	2.738196
H	0.060081	0.140033	1.661584
H	0.014581	2.971662	-2.106661
H	0.569880	1.455973	-2.866667
H	1.731218	2.811970	-2.628370
H	2.632115	-1.295436	-1.642942
H	4.442547	-0.246070	-0.414663

P: B + SF_2-MA
E = -3196.38
H = -3068.06
G = -3107.01
$\mathbf{N_{imag}}$ = 0

C	3.059205	-1.228103	-0.720405
C	3.615335	-0.108299	-0.245683
C	2.953981	0.769258	0.783258
C	1.698840	-1.728073	-0.309350
H	4.597307	0.199889	-0.611456
H	2.616288	1.708789	0.310418
H	3.693225	1.079645	1.537695
H	1.814835	-2.618186	0.332438
H	1.146922	-2.073018	-1.194291
C	1.779427	0.067022	1.475506
C	0.879614	-0.678864	0.461849
C	0.126416	0.327433	-0.397722
O	-0.577524	1.225185	0.052090
O	0.282722	0.146793	-1.724429
C	-0.440317	1.076268	-2.569712
S	-1.617282	1.688432	2.360533
F	-2.316200	2.070463	3.802773
F	-1.745521	0.061507	2.513211
H	2.166700	-0.683003	2.180061
H	1.181269	0.781340	2.054447
H	0.083708	-1.182888	1.031705
H	-0.105164	2.101786	-2.379075
H	-1.516457	1.007276	-2.375953
H	-0.206210	0.774229	-3.593179
H	3.602635	-1.830183	-1.451762

TS: B + SeF_2-MA
E = -3141.14
H = -3016.06
G = -3056.37
$\mathbf{N_{imag}}$ = 1, ν = -377i cm^{-1}

C	2.537724	-1.286564	-0.547982
C	3.594190	-0.682429	0.170402
C	3.500183	-0.381155	1.522579
C	1.354306	-1.657739	0.032968
H	4.337086	0.116330	2.011945

H	2.917370	-1.029145	2.175115
H	1.261523	-1.810260	1.104545
H	0.505285	-1.985670	-0.564875
C	2.026126	1.094276	1.650275
C	0.818432	0.680873	1.091688
C	0.350459	1.121249	-0.207848
O	-0.792436	0.946451	-0.667825
O	1.275303	1.816036	-0.919778
C	0.844450	2.283146	-2.217262
Se	-2.529232	-0.619158	-0.209088
F	-3.842919	-1.844186	0.025351
F	-2.084317	-0.615058	1.528715
H	2.594607	1.869452	1.142861
H	2.101738	1.096812	2.736886
H	0.061062	0.181481	1.689924
H	-0.009571	2.962566	-2.116210
H	0.557594	1.439305	-2.855624
H	1.707782	2.807145	-2.635164
H	2.620743	-1.319766	-1.635946
H	4.423648	-0.264494	-0.402885

P: B + SeF_2-MA
E = -3188.25
H = -3060.25
G = -3099.18
N_{imag} = 0

C	3.034591	-1.233109	-0.715607
C	3.589309	-0.108810	-0.250125
C	2.927133	0.774205	0.773284
C	1.675392	-1.733312	-0.300039
H	4.570723	0.198095	-0.618189
H	2.586039	1.709975	0.294797
H	3.666098	1.092630	1.524508
H	1.793349	-2.619788	0.346020
H	1.123210	-2.084709	-1.182543
C	1.758211	0.072605	1.473671
C	0.854235	-0.684268	0.469637
C	0.096232	0.308470	-0.394478
O	-0.661199	1.176642	0.042924
O	0.304508	0.172214	-1.713493
C	-0.417753	1.094168	-2.570057
Se	-1.577213	1.697987	2.282698
F	-2.256195	2.111217	3.898889
F	-1.564476	-0.063176	2.593839
H	2.148713	-0.674681	2.178920
H	1.162161	0.786439	2.055914
H	0.063835	-1.182768	1.050890
H	-0.133210	2.126639	-2.340184
H	-1.497378	0.974936	-2.428074
H	-0.124101	0.826374	-3.587453
H	3.578740	-1.840329	-1.442058

TS: B + TeF_2-MA
E = -3147.10
H = -3022.14

G = -3062.74
N$_{imag}$ = 1, ν = -371i cm^{-1}

C	2.534109	-1.293599	-0.550208
C	3.583074	-0.684832	0.176683
C	3.478642	-0.379287	1.528065
C	1.347624	-1.673323	0.016515
H	4.319015	0.107067	2.022736
H	2.889277	-1.026219	2.175932
H	1.233110	-1.801054	1.089269
H	0.510493	-2.014042	-0.591456
C	2.042977	1.110510	1.648954
C	0.824033	0.717788	1.097347
C	0.367463	1.149200	-0.203069
O	-0.777206	0.970647	-0.672532
O	1.289206	1.842727	-0.917085
C	0.864841	2.302712	-2.219181
Te	-2.593422	-0.619515	-0.297869
F	-3.963903	-1.969034	0.120725
F	-1.978355	-0.686885	1.552458
H	2.619021	1.878476	1.138650
H	2.118731	1.119493	2.735669
H	0.064741	0.217985	1.693455
H	0.014055	2.987275	-2.125555
H	0.577547	1.455990	-2.853339
H	1.732041	2.820377	-2.636883
H	2.632604	-1.337401	-1.636615
H	4.419713	-0.272422	-0.390139

P: B + TeF$_2$-MA
E = -3193.40
H = -3065.52
G = -3104.76
N$_{imag}$ = 0

C	3.030857	-1.247572	-0.712446
C	3.587691	-0.119735	-0.258309
C	2.924320	0.777033	0.752033
C	1.668482	-1.739045	-0.296595
H	4.571887	0.179228	-0.625330
H	2.586455	1.707965	0.261653
H	3.661181	1.103302	1.501893
H	1.781083	-2.620533	0.356977
H	1.117776	-2.096302	-1.177867
C	1.753271	0.085955	1.457864
C	0.847515	-0.682504	0.463156
C	0.090156	0.298515	-0.410791
O	-0.697509	1.150449	0.014657
O	0.330858	0.182409	-1.723327
C	-0.386424	1.098044	-2.591747
Te	-1.647772	1.791493	2.285006
F	-2.329063	2.093539	4.096436
F	-1.458556	-0.112322	2.642931
H	2.138909	-0.654109	2.172984
H	1.159818	0.808522	2.033152
H	0.058327	-1.171217	1.053942
H	-0.132627	2.133794	-2.341950

H	-1.466607	0.951308	-2.484702
H	-0.055161	0.848479	-3.602099
H	3.576023	-1.865599	-1.428900

TS: B + PF_3-MA
E = -3362.7
H = -3235.78
G = -3274.47
$\mathbf{N_{imag}}$ = 1, ν = -394i cm^{-1}

C	-1.649625	-0.648249	1.424949
C	-2.739141	-0.633958	0.527943
C	-2.775461	-1.434730	-0.604691
C	-0.563953	-1.470655	1.261660
H	-1.599474	0.144118	2.173857
H	-3.613397	-1.341770	-1.295130
H	-2.320295	-2.423280	-0.578196
H	-0.624007	-2.388350	0.683334
H	0.326876	-1.355788	1.876092
C	-1.165230	-0.752374	-1.794687
C	0.041803	-0.769851	-1.100428
C	0.699553	0.432894	-0.588139
O	1.871126	0.502401	-0.225402
O	-0.126605	1.523686	-0.579963
C	0.481394	2.738446	-0.094920
H	-1.613018	0.209176	-2.031435
H	-1.347690	-1.545719	-2.518410
H	0.697327	-1.636188	-1.148426
H	1.335328	3.019614	-0.722637
H	0.828616	2.612287	0.937675
H	-0.303117	3.498171	-0.148234
P	4.133539	-1.233976	0.209810
F	3.254072	-2.312153	-0.599147
F	5.371064	-2.221806	0.526628
F	3.406914	-1.397240	1.632165
H	-3.462957	0.177703	0.620228

P: B + PF_3-MA
E = -3411.77
H = -3282.92
G = -3320.44
$\mathbf{N_{imag}}$ = 0

C	2.674801	-1.679779	0.051906
C	3.204245	-0.988753	-0.963245
C	3.445665	0.496399	-0.912728
C	2.222060	-1.059377	1.347420
H	3.478394	-1.510332	-1.882830
H	2.712038	1.014677	-1.555612
H	4.428232	0.729487	-1.351281
H	2.902836	-1.361981	2.160894
H	1.232177	-1.454818	1.615108
C	3.378348	1.040253	0.519849
C	2.161770	0.474979	1.285697
C	0.830561	0.957349	0.710898
O	-0.130025	0.256675	0.450584

O	0.825141	2.307856	0.563081
C	-0.416111	2.871145	0.074417
P	-2.619252	-0.820499	-1.010671
F	-2.096669	0.181885	-2.150337
F	-3.363047	0.269231	-0.098511
F	-3.925321	-1.338474	-1.796184
H	3.354805	2.136152	0.520925
H	2.186227	0.881530	2.310123
H	-1.238990	2.632569	0.757799
H	-0.650250	2.475637	-0.920155
H	-0.246991	3.949994	0.032355
H	4.282429	0.731241	1.065217
H	2.542146	-2.759450	-0.045256

TS: B + AsF_3-MA
E = -3326.69
H = -3199.85
G = -3240.33
N_{imag} = 1, ν = -382i cm^{-1}

C	-1.573123	-0.671760	1.434656
C	-2.677891	-0.657744	0.554511
C	-2.732412	-1.450270	-0.583336
C	-0.486492	-1.486549	1.252914
H	-1.520515	0.111665	2.192896
H	-3.589802	-1.362445	-1.250113
H	-2.266396	-2.434094	-0.574075
H	-0.542344	-2.389440	0.651133
H	0.415093	-1.374055	1.851875
C	-1.177868	-0.734798	-1.809314
C	0.054909	-0.757387	-1.161169
C	0.709658	0.435127	-0.642110
O	1.896916	0.513736	-0.303319
O	-0.109248	1.522673	-0.594411
C	0.500473	2.732345	-0.095655
H	-1.638139	0.227598	-2.017348
H	-1.379055	-1.516954	-2.540186
H	0.709803	-1.622374	-1.231569
H	1.334081	3.036761	-0.739336
H	0.873398	2.584685	0.924706
H	-0.293082	3.484008	-0.112018
As	3.858866	-1.133290	0.236463
F	2.954124	-2.367492	-0.649938
F	5.174105	-2.242385	0.680591
F	3.002292	-1.330316	1.766775
H	-3.407509	0.146109	0.668289

P: B + AsF_3-MA
E = -3374.6
H = -3244.89
G = -3285.09
N_{imag} = 0

C	2.562104	-1.666047	0.092056
C	3.192208	-1.038156	-0.906393
C	3.507704	0.433611	-0.886017
C	2.062265	-0.979365	1.335897

H	3.497890	-1.604106	-1.788907
H	2.845010	0.965339	-1.591921
H	4.526854	0.601658	-1.266640
H	2.674327	-1.287674	2.200147
H	1.039184	-1.316822	1.554184
C	3.379996	1.029040	0.521630
C	2.088584	0.553104	1.226908
C	0.834840	1.092067	0.543648
O	-0.103210	0.413986	0.150507
O	0.869450	2.437814	0.455204
C	-0.292117	3.068003	-0.146577
As	-2.309920	-0.197382	-1.403608
F	-1.607479	0.896384	-2.594415
F	-3.161398	1.041923	-0.486389
F	-3.701573	-0.634821	-2.401907
H	3.413644	2.124291	0.488499
H	2.072255	0.993686	2.236653
H	-1.197678	2.809844	0.412826
H	-0.405084	2.742757	-1.186077
H	-0.093556	4.140994	-0.093976
H	4.229630	0.693434	1.134233
H	2.380038	-2.740490	0.020725

TS: B + SbF_3-MA
E = -3322.98
H = -3195.73
G = -3238.39
$\mathbf{N_{imag}}$ = 1, ν = -374i cm^{-1}

C	-1.544697	-0.684438	1.434680
C	-2.652089	-0.682335	0.555888
C	-2.703261	-1.471784	-0.584182
C	-0.448577	-1.484706	1.253612
H	-1.504863	0.094939	2.197968
H	-3.569398	-1.396959	-1.241154
H	-2.219701	-2.447161	-0.582543
H	-0.486853	-2.377962	0.635975
H	0.453467	-1.362305	1.850680
C	-1.186989	-0.717904	-1.823872
C	0.059678	-0.739351	-1.202540
C	0.705875	0.446357	-0.672773
O	1.904323	0.535089	-0.351123
O	-0.111779	1.527601	-0.592282
C	0.493162	2.731433	-0.071729
H	-1.659718	0.242901	-2.010828
H	-1.394256	-1.491387	-2.562250
H	0.717417	-1.601533	-1.284163
H	1.318984	3.056167	-0.715208
H	0.873189	2.563725	0.942727
H	-0.306822	3.476214	-0.066539
Sb	3.878098	-1.064829	0.195820
F	2.809129	-2.398321	-0.724275
F	5.147035	-2.422356	0.787904
F	2.898728	-1.172958	1.863646
H	-3.393519	0.109790	0.676535

P: B + SbF_3-MA

E = -3369.86

H = -3240.33

G = -3280.65

$\mathbf{N_{imag}}$ = 0

C	2.573060	-1.682570	0.160518
C	3.155842	-1.088912	-0.886393
C	3.446502	0.387017	-0.940127
C	2.108273	-0.951044	1.392251
H	3.440138	-1.687812	-1.754116
H	2.746666	0.878149	-1.639465
H	4.446197	0.554669	-1.369142
H	2.754843	-1.216794	2.245443
H	1.098922	-1.292697	1.662699
C	3.367555	1.038210	0.446049
C	2.111770	0.576422	1.224751
C	0.833389	1.082257	0.565869
O	-0.075196	0.369205	0.152709
O	0.811207	2.423547	0.516359
C	-0.368597	3.037075	-0.074680
Sb	-2.202205	-0.248579	-1.475629
F	-1.345750	1.036287	-2.641726
F	-3.120471	1.075791	-0.408412
F	-3.710487	-0.472379	-2.675426
H	3.383927	2.131753	0.368213
H	2.133721	1.057193	2.215189
H	-1.270838	2.711593	0.453476
H	-0.447228	2.761297	-1.131354
H	-0.213836	4.112381	0.038541
H	4.245353	0.738704	1.037086
H	2.407961	-2.761967	0.142937

Table S4. Cartesian coordinates (in Å), energies (in kcal mol^{-1}), and number of imaginary frequencies of all stationary points, computed at ZORA-BP86-D3(BJ)/TZ2P.

1,3-butadiene (B)
E = -1300.13
H = -1244.84
G = -1264.80
N_{imag} = 0

C	0.281823	0.581732	-0.869619
C	0.503176	-0.199146	0.196922
C	-0.529371	-0.807102	1.039267
C	-1.741522	-0.290776	1.284926
H	-0.728563	0.800503	-1.216560
H	1.105935	1.011287	-1.436603
H	1.536550	-0.437067	0.464845
H	-0.256514	-1.751158	1.519433
H	-2.462475	-0.813669	1.910996
H	-2.041998	0.676591	0.881166

HF
E = −181.91
H = −174.18
G = − 186.56
N_{imag} = 0

F	-1.097650	0.981173	0.000000
H	-0.165895	0.923022	0.000000

ClF
E = -93.75
H = -90.54
G = -106.07
N_{imag} = 0

Cl	-2.261078	-0.407459	-0.000001
F	-0.602473	-0.288958	-0.000000

BrF
E = -92.27
H = -89.19
G = -105.52
N_{imag} = 0

Cl	-2.261078	-0.407459	-0.000001
F	-0.602473	-0.288958	-0.000000

IF
E = −96.67
H = −93.65
G = −110.50
N_{imag} = 0

I	-1.140026	-0.163810	0.000070
F	-3.059542	0.152359	-0.000129

SF_2
E = −244.74
H = −239.33

G = -257.80
N$_{imag}$ = 0

S	0.160282	-0.118824	0.000000
F	-1.058554	-1.193543	0.000000
F	1.422462	-1.142235	0.000000

SeF$_2$
E = -232.93
H = -227.89
G = -247.36
N$_{imag}$ = 0

Se	-0.560460	-0.335950	0.004078
F	0.266654	0.463289	-1.340191
F	0.267255	0.492769	1.329801

TeF$_2$
E = -236.05
H = -231.13
G = -251.28
N$_{imag}$ = 0

Te	-0.623904	-0.398365	0.004689
F	0.298323	0.493437	-1.433603
F	0.299030	0.525035	1.422602

PF$_3$
E = -462.88
H = -454.69
G = -474.34
N$_{imag}$ = 0

P	-0.490016	-0.184315	-0.022576
F	-1.754220	-1.093825	-0.390349
F	0.645689	-1.144794	-0.613132
F	-0.583048	0.825763	-1.260262

AsF$_3$
E = -422.47
H = -415.05
G = -435.91
N$_{imag}$ = 0

As	0.019824	0.010283	-0.664716
F	1.503551	0.010244	0.266035
F	-0.748954	-1.318011	0.179907
F	-0.774422	1.297484	0.218774

SbF$_3$
E = -415.53
H = -408.35
G = -430.15
N$_{imag}$ = 0

Sb	0.021655	0.011256	-0.750188
F	1.631036	0.011448	0.298590
F	-0.812837	-1.431427	0.204570
F	-0.839853	1.408723	0.247028

Methyl acrylate (MA)
E = −1626.26

H = -1563.66
G = - 1587.82
$\mathbf{N_{imag}}$ **=** 0

O	1.271085	0.798317	-0.617623
C	2.119164	1.844965	-0.098385
H	-0.560938	-0.268398	-1.910315
H	-0.930739	-2.095837	-1.944996
H	0.955803	-2.504040	-0.402960
H	3.160545	1.676935	-0.396989
H	2.063880	1.874354	0.996138
H	1.735516	2.771873	-0.531686
O	2.518288	-0.687565	0.570078
C	-0.316416	-1.275373	-1.576435
C	0.702620	-1.499995	-0.742982
C	1.593761	-0.453954	-0.188773

HF-MA
E = -1819.65
H = -1747.77
G = -1775.77
$\mathbf{N_{imag}}$ = 0

C	3.703015	2.468717	0.000095
C	2.704233	1.581347	0.000122
C	2.898114	0.118789	-0.000076
O	1.969847	-0.692181	-0.000051
O	4.185328	-0.269978	-0.000285
C	4.399394	-1.702893	-0.000482
H	4.744699	2.152144	-0.000073
H	3.497841	3.538049	0.000251
H	1.657048	1.884716	0.000296
H	3.951350	-2.151566	-0.893851
H	3.951552	-2.151782	0.892880
H	5.484007	-1.829701	-0.000620
H	0.480502	-0.082407	0.000233
F	-0.367109	0.384127	0.000412

ClF-MA
E = -1730.90
H = -1664.02
G = -1694.30
$\mathbf{N_{imag}}$ = 0

C	3.902941	2.454568	0.000391
C	2.905425	1.565614	0.000554
C	3.106657	0.107604	-0.000080
O	2.192288	-0.724480	-0.000001
O	4.393753	-0.276825	-0.000770
C	4.617940	-1.709657	-0.001410
H	4.945547	2.141032	-0.000219
H	3.694801	3.523281	0.000864
H	1.858649	1.865693	0.001166
H	4.172656	-2.160172	-0.895044
H	4.173486	-2.160838	0.892302
H	5.703463	-1.827085	-0.001956
Cl	0.003491	-0.075073	0.001015
F	-1.659587	0.376860	0.001771

BrF-MA
E = -1732.52
H = -1665.77
G = -1696.79
$\mathbf{N_{imag}}$ = 0

C	3.957423	2.451516	-0.010844
C	2.951612	1.571911	-0.006051
C	3.139315	0.113350	0.000266
O	2.215678	-0.711324	0.004953
O	4.419517	-0.286379	0.000697
C	4.629678	-1.721998	0.006965
H	4.997139	2.128414	-0.010336
H	3.758956	3.522014	-0.015391
H	1.907044	1.881650	-0.006465
H	4.179730	-2.171901	-0.884536
H	4.181309	-2.163875	0.903259
H	5.713983	-1.849385	0.006574
Br	-0.050817	-0.094745	0.004815
F	-1.849433	0.348367	0.004960

IF-MA
E = -1737.97
H = -1671.28
G = -1702.96
$\mathbf{N_{imag}}$ = 0

C	4.012779	2.461924	-0.009098
C	3.000506	1.589681	-0.008154
C	3.175560	0.129836	-0.001126
O	2.243151	-0.685259	0.001818
O	4.451220	-0.281926	0.001983
C	4.646222	-1.719838	0.009630
H	5.050010	2.130807	-0.004683
H	3.822757	3.533955	-0.014253
H	1.958118	1.909219	-0.012186
H	4.193362	-2.165229	-0.882622
H	4.190761	-2.156088	0.905066
H	5.729051	-1.858799	0.011889
I	-0.186231	-0.136946	0.004665
F	-2.136097	0.266333	0.008231

SF_2–MA
E = -1877.69
H = -1808.42
G = -1842.02
$\mathbf{N_{imag}}$ = 0

S	-3.190290	0.348537	-0.251526
F	-2.491093	0.519048	1.219912
F	-4.584862	-0.351844	0.290118
O	1.171443	1.538853	-1.265404
C	1.008488	2.908021	-1.706673
H	2.339884	-0.519654	-0.511394
H	1.495220	-2.092750	0.024720
H	-0.709594	-1.024739	-0.309692
H	0.511933	3.499617	-0.929614
H	0.408823	2.941849	-2.623097
H	2.021611	3.272696	-1.889481

O	-1.075196	1.433550	-1.073228
C	1.415959	-1.063423	-0.321609
C	0.222002	-0.492417	-0.501370
C	0.024878	0.895147	-0.962772

SeF2-MA
E = -1869.86
H = -1801.48
G = -1833.87
$\mathbf{N_{imag}}$ = 0

Se	-3.155500	0.372441	-0.384455
F	-2.554490	0.190383	1.289523
F	-4.670691	-0.556064	-0.080100
O	1.175763	1.572113	-1.219482
C	1.015816	2.916208	-1.736675
H	2.247832	-0.655102	-0.915496
H	1.456289	-2.099327	-0.041696
H	-0.628233	-0.808300	0.221643
H	0.652549	3.582630	-0.946805
H	0.304467	2.919784	-2.569754
H	2.012194	3.212708	-2.071066
O	-1.033668	1.573673	-0.783226
C	1.378832	-1.079869	-0.416104
C	0.246758	-0.385957	-0.268809
C	0.052792	0.987066	-0.768032

TeF2-MA
E = -1876.38
H = -1808.11
G = -1840.74
$\mathbf{N_{imag}}$ = 0

C	1.739493	1.509856	1.807382
C	0.620489	1.080104	1.214315
C	0.303223	1.317968	-0.199800
O	-0.701044	0.871667	-0.771013
O	1.201139	2.070003	-0.859409
C	0.915769	2.303920	-2.259816
Te	-2.583502	-0.707777	-0.296616
F	-3.995548	-1.965135	0.214162
F	-2.046545	-0.525404	1.567368
H	2.491782	2.079188	1.264100
H	1.927220	1.295266	2.858107
H	-0.138328	0.505506	1.743237
H	-0.031349	2.844189	-2.366914
H	0.854236	1.351685	-2.797997
H	1.751151	2.905245	-2.624374

PF_3-MA
E = -2093.39
H = -2021.94
G = -2055.99
$\mathbf{N_{imag}}$ = 0

F	-1.705156	0.745374	1.428036
H	0.177234	-3.221071	-2.004833
H	0.597101	-3.311347	-0.272203
F	-4.035955	1.240933	1.144326

H	1.868723	-3.605153	-1.515194
H	2.866135	0.313404	-1.498163
H	2.340389	2.090260	-1.292186
H	0.032560	1.354994	-0.823528
C	2.077090	1.033540	-1.288675
C	0.826243	0.640377	-1.033935
C	0.379952	-0.768877	-1.006160
O	-0.766078	-1.117287	-0.753404
O	1.368533	-1.651436	-1.292027
C	0.967441	-3.039693	-1.267357
P	-2.900479	0.342632	0.433032
F	-2.555356	1.389754	-0.738343

AsF_3-MA
E = -2057.26
H = -1986.52
G = -2020.85
$\mathbf{N_{imag}}$ = 0

F	-1.522878	0.900112	1.456693
H	0.101661	-3.203207	-1.993667
H	0.613964	-3.329439	-0.286617
F	-4.070029	1.250489	1.095921
H	1.817656	-3.589643	-1.602525
H	2.822591	0.314824	-1.518927
H	2.309646	2.088340	-1.260678
H	0.020222	1.351293	-0.710174
C	2.044088	1.032363	-1.265630
C	0.802490	0.639953	-0.966742
C	0.363553	-0.768277	-0.943942
O	-0.767130	-1.133246	-0.625701
O	1.323184	-1.644021	-1.310516
C	0.928406	-3.035376	-1.294260
As	-2.779316	0.252808	0.403340
F	-2.473796	1.347651	-0.946467

SbF_3-MA
E = -2053.65
H = -1983.09
G = -2017.83
$\mathbf{N_{imag}}$ = 0

F	-1.359699	0.486730	1.528899
H	0.194673	-3.246947	-1.994938
H	0.500750	-3.264120	-0.233653
F	-4.012849	1.336995	1.312531
H	1.853857	-3.587685	-1.379250
H	2.830408	0.329029	-1.334447
H	2.276176	2.106879	-1.252536
H	-0.070489	1.370357	-1.013236
C	2.019169	1.048943	-1.240954
C	0.747774	0.658982	-1.110922
C	0.321490	-0.750043	-1.078369
O	-0.845126	-1.115338	-0.903853
O	1.317516	-1.634487	-1.265737
C	0.930722	-3.028317	-1.213448
Sb	-2.825164	0.168902	0.310413
F	-2.338627	1.592334	-0.910904

RC: B + MA
E = -2930.32
H = -2811.21
G = -2846.86
$\mathbf{N_{imag}}$ = 0

C	0.077466	-0.164581	1.640452
C	-1.282479	0.241890	1.293451
C	-2.327167	-0.575366	1.091820
C	0.611496	-1.380177	1.449625
H	-1.441846	1.318744	1.197565
H	-3.303723	-0.183183	0.813904
H	-2.245276	-1.655143	1.216934
H	0.044945	-2.189980	0.991389
H	1.640796	-1.594534	1.729012
C	-1.122954	-1.126570	-1.969558
C	0.193257	-1.324280	-1.848572
C	1.186319	-0.260665	-1.584414
O	2.388166	-0.453204	-1.517312
O	0.614111	0.967675	-1.432076
C	1.547808	2.023438	-1.131807
H	-1.554791	-0.133222	-1.875358
H	-1.799798	-1.956648	-2.167366
H	0.638043	-2.314347	-1.945261
H	2.279492	2.133619	-1.940733
H	2.081371	1.804471	-0.199068
H	0.943079	2.928133	-1.029950
H	0.711885	0.619164	2.062118

TS: B + MA
E = -2921.96
H = -2803.17
G = -2833.72
$\mathbf{N_{imag}}$ = 1, n = -371i cm^{-1}

C	-0.022989	-0.226121	1.546382
C	-1.368796	0.087953	1.265483
C	-2.191276	-0.769367	0.555758
C	0.555644	-1.404119	1.145511
H	-1.695199	1.116436	1.426091
H	-3.199603	-0.453852	0.291394
H	-2.056895	-1.845726	0.641670
H	-0.039202	-2.294178	0.960897
H	1.629414	-1.558643	1.227917
C	-1.238141	-0.894029	-1.383492
C	0.108372	-1.207968	-1.256239
C	1.178661	-0.209754	-1.361343
O	2.363042	-0.454913	-1.530840
O	0.692261	1.070176	-1.254751
C	1.702878	2.093855	-1.310393
H	-1.513532	0.121477	-1.651908
H	-1.926784	-1.675806	-1.699222
H	0.455513	-2.232796	-1.364214
H	2.240127	2.055870	-2.265455
H	2.426249	1.965891	-0.495624
H	1.166984	3.041035	-1.205464
H	0.617768	0.572974	1.921507

P: B + MA
E = -2970.67
H = -2848.93
G = -2878.51
N_{imag} = 0

C	2.685175	-1.948372	-0.372741
C	3.928403	-1.504594	-0.161736
C	4.247981	-0.343263	0.739983
C	1.456565	-1.318355	0.225627
H	4.765461	-1.995266	-0.662470
H	4.538431	0.531392	0.131612
H	5.131864	-0.578246	1.352127
H	1.030340	-1.985534	0.993088
H	0.687966	-1.208947	-0.551620
C	3.067270	0.015373	1.648020
C	1.748484	0.051825	0.851057
C	1.744374	1.145241	-0.213586
O	1.365681	1.027122	-1.360755
O	2.195570	2.325074	0.300722
C	2.190043	3.428963	-0.631081
H	3.238818	0.974003	2.149931
H	2.963125	-0.753460	2.427570
H	0.932202	0.321730	1.540480
H	1.170384	3.630817	-0.979525
H	2.822032	3.200610	-1.497126
H	2.586285	4.282003	-0.074657
H	2.525956	-2.807088	-1.027664

RC: B + HF-MA
E = -3124.83
H = -2996.42
G = -3035.37
N_{imag} = 0

C	2.737930	-1.207995	-1.258638
C	3.878549	-1.352879	-0.358844
C	3.995213	-2.230167	0.651708
C	1.543008	-1.808985	-1.136780
H	2.881839	-0.515432	-2.091508
H	4.883567	-2.247522	1.280137
H	3.226579	-2.972226	0.866213
H	1.322801	-2.502458	-0.326388
H	0.738998	-1.621137	-1.844667
C	1.916129	-0.636811	2.437821
C	0.920156	-0.318100	1.602756
C	0.976712	0.803439	0.652577
O	0.043879	1.131003	-0.086095
O	2.143822	1.473045	0.660638
C	2.237743	2.556487	-0.292331
H	2.841966	-0.067480	2.460372
H	1.819552	-1.473292	3.127862
H	-0.016922	-0.873274	1.573987
H	1.475366	3.315114	-0.083869
H	2.097216	2.174851	-1.309876
H	3.241980	2.966013	-0.163218
H	-1.172328	0.114356	-0.158993
F	-1.852613	-0.579032	-0.170018

H	4.706042	-0.661687	-0.535693

TS: B + HF-MA
E = -3117.91
H = -2989.85
G = -3023.97
$\mathbf{N_{imag}}$ = 1, n = -345i cm^{-1}

C	2.839119	-1.234323	-1.302202
C	3.888044	-1.484604	-0.393274
C	3.693977	-2.191599	0.783471
C	1.560406	-1.683729	-1.092048
H	3.018868	-0.511906	-2.099758
H	4.521990	-2.305570	1.481761
H	2.957164	-2.991994	0.815462
H	1.353122	-2.535143	-0.450371
H	0.729851	-1.352278	-1.711456
C	2.351079	-0.969680	1.902808
C	1.227638	-0.643328	1.150100
C	1.080884	0.635854	0.474279
O	0.034078	1.068230	-0.029584
O	2.223444	1.364445	0.446555
C	2.131947	2.621395	-0.258299
H	3.090274	-0.197925	2.096873
H	2.246809	-1.730891	2.673872
H	0.308389	-1.219958	1.220322
H	1.391761	3.274429	0.217254
H	1.841238	2.453132	-1.301754
H	3.131540	3.058550	-0.198737
H	-1.107283	0.013224	-0.152858
F	-1.742301	-0.724766	-0.239824
H	4.818958	-0.931188	-0.525364

P: B + HF-MA
E = -3163.54
H = -3032.48
G = -3065.83
$\mathbf{N_{imag}}$ = 0

C	-3.160111	-1.901563	0.375430
C	-4.402164	-1.421776	0.250605
C	-4.798509	-0.384116	-0.761617
C	-1.998827	-1.436225	-0.458963
H	-5.188088	-1.793160	0.911627
H	-5.097790	0.538818	-0.240535
H	-5.698262	-0.722539	-1.298525
H	-1.777177	-2.175171	-1.245964
H	-1.086092	-1.374729	0.146252
C	-3.682193	-0.087282	-1.774132
C	-2.286356	-0.077036	-1.131661
C	-2.035324	1.060545	-0.156565
O	-1.017873	1.186700	0.520417
O	-3.018443	1.973569	-0.115781
C	-2.791761	3.094366	0.776872
H	-3.879206	0.857904	-2.294134
H	-3.670437	-0.877733	-2.538281
H	-1.525790	0.077851	-1.914354
H	-1.885459	3.633999	0.482282

H	-2.682073	2.737046	1.806278
H	-3.676750	3.725650	0.672747
H	0.315314	0.342993	0.244140
F	1.106647	-0.169619	0.029384
H	-2.953439	-2.673166	1.119229

RC: B + ClF-MA
E = -3036.78
H = -2913.33
G = -2953.83
$\mathbf{N_{imag}}$ = 0

C	2.715978	-1.218240	-1.268353
C	3.847985	-1.401064	-0.365603
C	3.940747	-2.296240	0.632279
C	1.517427	-1.818748	-1.180460
H	2.870037	-0.497092	-2.074628
H	4.826063	-2.343115	1.263457
H	3.156512	-3.026238	0.830806
H	1.287966	-2.544848	-0.401638
H	0.720813	-1.599669	-1.887556
C	1.915844	-0.709595	2.409920
C	0.906408	-0.393358	1.589133
C	0.946286	0.738309	0.657360
O	0.016430	1.079938	-0.087412
O	2.097246	1.431857	0.677401
C	2.176468	2.532942	-0.258878
H	2.835409	-0.130392	2.430872
H	1.834181	-1.553283	3.092913
H	-0.024468	-0.956367	1.565031
H	1.408333	3.280380	-0.032715
H	2.034392	2.166479	-1.281569
H	3.177600	2.948394	-0.126037
Cl	-1.778928	-0.257808	-0.338878
F	-3.156952	-1.276947	-0.588340
H	4.690216	-0.723937	-0.526347

TS: B + ClF-MA
E = -3030.40
H = -2907.27
G = -2943.20
$\mathbf{N_{imag}}$ = 1, n = -331i cm^{-1}

C	2.843537	-1.236000	-1.301100
C	3.894905	-1.487953	-0.394979
C	3.706152	-2.196765	0.782212
C	1.566101	-1.688439	-1.095357
H	3.023249	-0.514963	-2.099881
H	4.540864	-2.318214	1.471176
H	2.967265	-2.995273	0.816511
H	1.355645	-2.534194	-0.447150
H	0.736867	-1.353935	-1.714882
C	2.400808	-0.968706	1.915257
C	1.255370	-0.655080	1.189846
C	1.093453	0.607427	0.501049
O	0.043222	1.046577	-0.008335
O	2.222350	1.352218	0.454520
C	2.115285	2.599808	-0.268128

H	3.139727	-0.191170	2.086457
H	2.316118	-1.720521	2.697947
H	0.345118	-1.240675	1.280981
H	1.379444	3.256077	0.209252
H	1.811462	2.414310	-1.304659
H	3.114186	3.040209	-0.227135
Cl	-1.694887	-0.320501	-0.179379
F	-3.064210	-1.384200	-0.367254
H	4.826916	-0.937491	-0.531563

P: B + ClF-MA
E = -3075.29
H = -2949.16
G = -2984.26
$\mathbf{N_{imag}}$ = 0

C	-3.212853	-1.903227	0.288624
C	-4.460761	-1.525802	-0.007061
C	-4.797283	-0.477719	-1.028000
C	-1.986691	-1.312837	-0.348746
H	-5.298162	-1.993261	0.515126
H	-5.259886	0.383559	-0.522594
H	-5.568942	-0.867871	-1.709820
H	-1.581750	-2.002937	-1.106449
H	-1.184805	-1.191253	0.390698
C	-3.578457	-0.028016	-1.848755
C	-2.275742	0.044533	-1.037600
C	-2.200037	1.156111	-0.016142
O	-1.173572	1.482655	0.587744
O	-3.351496	1.801543	0.205076
C	-3.297869	2.871763	1.185597
H	-3.781190	0.933604	-2.337050
H	-3.399855	-0.758390	-2.650951
H	-1.432911	0.253391	-1.713260
H	-2.571407	3.629986	0.875312
H	-3.009643	2.468852	2.162102
H	-4.309415	3.281900	1.212898
Cl	0.848975	0.700397	-0.087599
F	2.406526	0.159841	-0.603184
H	-3.049152	-2.686627	1.030907

RC: B + BrF-MA
E = -3038.77
H = -2915.46
G = -2956.54
$\mathbf{N_{imag}}$ = 0

C	2.719195	-1.223821	-1.287256
C	3.884160	-1.382997	-0.423080
C	4.024386	-2.269507	0.577132
C	1.532366	-1.839048	-1.151612
H	2.835907	-0.510288	-2.106513
H	4.931673	-2.299373	1.177361
H	3.258724	-3.010030	0.806699
H	1.339244	-2.559424	-0.357753
H	0.709424	-1.640078	-1.834125
C	2.070583	-0.697170	2.421308
C	1.029950	-0.392706	1.635433

C	1.029813	0.730659	0.695308
O	0.069972	1.062631	-0.018600
O	2.173662	1.431881	0.665387
C	2.212749	2.523795	-0.284910
H	2.986185	-0.111560	2.405853
H	2.018722	-1.536350	3.112641
H	0.102090	-0.962023	1.648377
H	1.446254	3.267263	-0.041227
H	2.040516	2.144377	-1.298138
H	3.214281	2.948196	-0.189253
Br	-1.815858	-0.267526	-0.239411
F	-3.329809	-1.321418	-0.477746
H	4.710500	-0.695323	-0.617885

TS: B + BrF-MA
E = -3032.60
H = -2909.60
G = -2946.24
$\mathbf{N_{imag}}$ = 1, n = -327i cm^{-1}

C	2.856145	-1.234719	-1.301121
C	3.907177	-1.479707	-0.392506
C	3.721765	-2.192117	0.783383
C	1.582022	-1.698173	-1.101047
H	3.033280	-0.512950	-2.099821
H	4.557459	-2.310576	1.471672
H	2.988824	-2.996274	0.814374
H	1.374648	-2.543060	-0.450728
H	0.752275	-1.371443	-1.724344
C	2.416038	-0.975729	1.918890
C	1.265930	-0.667820	1.197931
C	1.095961	0.590521	0.507795
O	0.040375	1.024066	-0.000745
O	2.217068	1.343828	0.455766
C	2.099797	2.588754	-0.270709
H	3.149323	-0.192760	2.090157
H	2.337352	-1.728038	2.701768
H	0.359826	-1.260979	1.288335
H	1.358538	3.240130	0.204794
H	1.798017	2.397353	-1.306728
H	3.095111	3.037079	-0.230595
Br	-1.795839	-0.323708	-0.183433
F	-3.304551	-1.409357	-0.390697
H	4.835732	-0.922907	-0.526888

P: B + BrF-MA
E = -3077.12
H = -2951.11
G = -2986.93
$\mathbf{N_{imag}}$ = 0

C	-3.252632	-1.901625	0.252910
C	-4.491658	-1.516681	-0.069001
C	-4.800197	-0.458352	-1.088054
C	-2.009727	-1.311428	-0.350694
H	-5.342910	-1.985492	0.428920
H	-5.274423	0.398636	-0.586223
H	-5.554900	-0.840579	-1.792894

H	-1.590276	-1.995018	-1.106257
H	-1.222713	-1.202643	0.407154
C	-3.560679	-0.003025	-1.874180
C	-2.275898	0.054324	-1.033725
C	-2.215567	1.154246	-0.001226
O	-1.190230	1.493063	0.602098
O	-3.373996	1.775873	0.237507
C	-3.334609	2.832970	1.233800
H	-3.748216	0.965410	-2.355161
H	-3.365775	-0.723701	-2.681229
H	-1.415944	0.263738	-1.687843
H	-2.626757	3.610492	0.928542
H	-3.030394	2.421426	2.201709
H	-4.354262	3.221057	1.274533
Br	0.928398	0.724011	-0.016065
F	2.631432	0.171013	-0.504506
H	-3.109202	-2.691951	0.991971

RC: B + IF-MA
E = -3044.62
H = -2921.37
G = -2962.91
$\mathbf{N_{imag}}$ = 0

C	2.700230	-1.236661	-1.281895
C	3.883017	-1.355932	-0.435938
C	4.064139	-2.229560	0.569053
C	1.533400	-1.884091	-1.122723
H	2.783697	-0.528150	-2.109531
H	4.981742	-2.229310	1.154154
H	3.323405	-2.989231	0.817147
H	1.372529	-2.601927	-0.319345
H	0.695573	-1.716010	-1.795493
C	2.097287	-0.704781	2.435700
C	1.058092	-0.395765	1.649614
C	1.060100	0.728965	0.711882
O	0.097912	1.065430	0.002860
O	2.206805	1.424199	0.677434
C	2.244640	2.519533	-0.269213
H	3.013672	-0.120319	2.422979
H	2.043306	-1.545906	3.124513
H	0.128920	-0.964324	1.661378
H	1.482595	3.264973	-0.017878
H	2.064337	2.144621	-1.282656
H	3.248450	2.939437	-0.178178
I	-1.954579	-0.248784	-0.308813
F	-3.621210	-1.303809	-0.622998
H	4.686749	-0.647621	-0.650275

TS: B + IF-MA
E = -3038.46
H = -2915.53
G = -2952.71
$\mathbf{N_{imag}}$ = 1, n = -325i cm^{-1}

C	2.868901	-1.234151	-1.299023
C	3.924863	-1.473616	-0.394882
C	3.747329	-2.185350	0.783108

C	1.597689	-1.703107	-1.093704
H	3.039998	-0.514240	-2.100691
H	4.587656	-2.301630	1.466122
H	3.018392	-2.993085	0.817072
H	1.395247	-2.545735	-0.438895
H	0.764528	-1.383333	-1.716459
C	2.450221	-0.974607	1.924518
C	1.294512	-0.666440	1.211900
C	1.118578	0.590294	0.521712
O	0.056827	1.021761	0.023826
O	2.239142	1.342782	0.456494
C	2.113847	2.586264	-0.271195
H	3.182257	-0.189683	2.092690
H	2.376352	-1.726068	2.708763
H	0.389572	-1.261605	1.309223
H	1.377023	3.238023	0.210571
H	1.801469	2.392606	-1.303621
H	3.109245	3.035205	-0.242176
I	-1.949353	-0.321998	-0.209292
F	-3.610216	-1.416380	-0.464591
H	4.851021	-0.914209	-0.534692

P: B + IF-MA
E = -3082.85
H = -2956.91
G = -2993.32
$\mathbf{N_{imag}}$ = 0

C	-3.273863	-1.907521	0.249023
C	-4.513798	-1.520687	-0.067105
C	-4.825418	-0.460526	-1.083316
C	-2.033199	-1.316972	-0.358596
H	-5.363480	-1.989571	0.433391
H	-5.295954	0.396636	-0.578302
H	-5.583925	-0.840610	-1.785173
H	-1.616097	-1.999029	-1.116807
H	-1.242778	-1.211144	0.396807
C	-3.588711	-0.006255	-1.874437
C	-2.301790	0.050040	-1.037487
C	-2.237491	1.148610	-0.004135
O	-1.208721	1.481892	0.596930
O	-3.391789	1.775966	0.235444
C	-3.344219	2.834361	1.230310
H	-3.777055	0.962461	-2.354440
H	-3.397221	-0.727075	-2.682137
H	-1.442979	0.260820	-1.694155
H	-2.633608	3.607876	0.921412
H	-3.038415	2.422550	2.197555
H	-4.361839	3.227300	1.273848
I	1.082544	0.751808	0.002340
F	2.943942	0.224971	-0.481255
H	-3.127960	-2.699547	0.985726

RC: B + SF_2-MA
E = -3182.93
H = -3057.09
G = -3100.96

N$_{imag}$ = 0

C	2.221763	-1.202975	-0.526360
C	3.440761	-0.586220	-0.011840
C	3.819797	-0.509982	1.274683
C	1.212954	-1.711685	0.199935
H	4.744094	-0.010553	1.558616
H	3.238344	-0.961028	2.078315
H	1.233063	-1.725326	1.288971
H	0.325637	-2.123017	-0.276900
C	1.559012	1.602250	1.981676
C	0.472782	1.166507	1.332373
C	0.245564	1.359682	-0.107896
O	-0.767874	1.004741	-0.709449
O	1.258759	1.997219	-0.735832
C	1.082635	2.170477	-2.158639
S	-2.439632	-0.771598	-0.380251
F	-3.535304	-2.007059	-0.317592
F	-2.157682	-0.717306	1.237017
H	2.352489	2.136971	1.465771
H	1.671278	1.439759	3.052384
H	-0.332541	0.639094	1.838242
H	0.197241	2.782891	-2.363156
H	0.964913	1.195872	-2.646100
H	1.990482	2.671346	-2.502495
H	2.127170	-1.218790	-1.614825
H	4.090364	-0.133560	-0.764673

TS: B + SF$_2$-MA
E = -3175.90
H = -3050.39
G = -3089.39
N$_{imag}$ = 1, n = -343i cm^{-1}

C	2.385277	-1.201850	-0.568905
C	3.503370	-0.633311	0.075076
C	3.519616	-0.377114	1.437840
C	1.246003	-1.556803	0.107511
H	4.383558	0.118595	1.878433
H	2.977379	-1.034144	2.115208
H	1.258704	-1.764540	1.173668
H	0.335422	-1.841437	-0.417301
C	1.998431	1.089211	1.705894
C	0.798416	0.679367	1.132749
C	0.361005	1.134367	-0.176739
O	-0.767632	0.987754	-0.662721
O	1.335878	1.789012	-0.863780
C	0.966997	2.214785	-2.191895
S	-2.394469	-0.736661	-0.258464
F	-3.494032	-1.970433	-0.103085
F	-2.149057	-0.552747	1.357594
H	2.565893	1.876696	1.218725
H	2.089259	1.040859	2.789646
H	0.024290	0.200522	1.723979
H	0.128123	2.918636	-2.150767
H	0.678182	1.352012	-2.803715
H	1.859161	2.697197	-2.598678
H	2.367634	-1.193280	-1.659792

H	4.285807	-0.200499	-0.549978

P: B + SF_2-MA
E = -3222.46
H = -3093.98
G = -3132.19
$\mathbf{N_{imag}}$ = 0

C	3.009263	-1.213301	-0.718762
C	3.547682	-0.076570	-0.265881
C	2.889674	0.791846	0.771696
C	1.669567	-1.737162	-0.275172
H	4.512696	0.252585	-0.656502
H	2.515725	1.718167	0.300331
H	3.637748	1.125729	1.506320
H	1.813770	-2.610793	0.382347
H	1.109133	-2.107485	-1.143925
C	1.748419	0.066869	1.491049
C	0.848327	-0.688106	0.487731
C	0.123563	0.317831	-0.387851
O	-0.572554	1.233874	0.040318
O	0.304045	0.129982	-1.707665
C	-0.373738	1.078500	-2.567285
S	-1.510619	1.653041	2.315940
F	-2.166366	1.991625	3.793074
F	-1.641683	0.021437	2.438407
H	2.163424	-0.679317	2.182917
H	1.148737	0.768920	2.082317
H	0.046928	-1.182114	1.056182
H	-0.012068	2.093083	-2.367463
H	-1.454809	1.040980	-2.394541
H	-0.126722	0.769417	-3.585211
H	3.549766	-1.807675	-1.457826

RC: B + SeF_2-MA
E = -3176.18
H = -3051.23
G = -3093.58
$\mathbf{N_{imag}}$ = 0

C	2.396265	-1.266594	-0.609009
C	3.571997	-0.648709	-0.003388
C	3.892105	-0.643227	1.301417
C	1.351341	-1.806723	0.039993
H	4.787165	-0.136637	1.657371
H	3.292442	-1.164071	2.047319
H	1.300552	-1.847071	1.127060
H	0.503677	-2.223046	-0.500541
C	1.651742	1.437773	1.999346
C	0.564713	0.982626	1.363474
C	0.311146	1.196516	-0.065240
O	-0.705205	0.823014	-0.662424
O	1.284388	1.874912	-0.703521
C	1.081213	2.060033	-2.122208
Se	-2.514368	-0.706046	-0.231461
F	-3.889661	-1.857769	-0.031694
F	-1.898203	-0.982383	1.428800
H	2.419044	2.006369	1.480250

H	1.788150	1.259305	3.064542
H	-0.214904	0.419325	1.870982
H	0.170642	2.641062	-2.305703
H	0.995518	1.087179	-2.619538
H	1.964485	2.599862	-2.470472
H	2.370999	-1.254475	-1.701446
H	4.240948	-0.133948	-0.697206

TS: B + SeF_2-MA
E = -3169.62
H = -3044.40
G = -3084.23
N_{imag} = 1, n = -333i cm^{-1}

C	2.433310	-1.186239	-0.579831
C	3.527976	-0.603568	0.092102
C	3.515024	-0.359819	1.457931
C	1.287509	-1.572148	0.066929
H	4.366129	0.140453	1.918045
H	2.971962	-1.033232	2.118470
H	1.274522	-1.781378	1.132536
H	0.396889	-1.872956	-0.482850
C	1.991445	1.087454	1.710977
C	0.795857	0.664148	1.137039
C	0.363105	1.103117	-0.173019
O	-0.764138	0.931219	-0.669624
O	1.316981	1.777616	-0.862520
C	0.946021	2.179022	-2.198491
Se	-2.496990	-0.621223	-0.229694
F	-3.829494	-1.829220	-0.007457
F	-1.997359	-0.716070	1.491508
H	2.548752	1.883962	1.226317
H	2.076277	1.043807	2.795462
H	0.029019	0.163663	1.720015
H	0.079306	2.848687	-2.171850
H	0.701068	1.300030	-2.806347
H	1.822853	2.693921	-2.598125
H	2.441304	-1.170888	-1.670814
H	4.315606	-0.152700	-0.513448

P: B + SeF_2-MA
E = -3215.37
H = -3087.21
G = -3125.77
N_{imag} = 0

C	2.989852	-1.229146	-0.720358
C	3.534709	-0.088915	-0.284555
C	2.885000	0.793576	0.746233
C	1.650863	-1.743821	-0.263665
H	4.499033	0.231780	-0.683585
H	2.508508	1.714600	0.265882
H	3.638232	1.136744	1.471081
H	1.795462	-2.613068	0.399196
H	1.083189	-2.119205	-1.125829
C	1.750413	0.078168	1.483905
C	0.837588	-0.688405	0.498875
C	0.107046	0.305725	-0.378694

O	-0.632147	1.198602	0.041668
O	0.327352	0.156292	-1.692084
C	-0.352036	1.101693	-2.556133
Se	-1.537087	1.664550	2.260480
F	-2.235357	2.026480	3.883232
F	-1.525154	-0.104891	2.528730
H	2.169349	-0.662991	2.178352
H	1.158682	0.785392	2.077570
H	0.042941	-1.170916	1.085370
H	-0.026954	2.121952	-2.325778
H	-1.436050	1.026796	-2.418795
H	-0.062661	0.818920	-3.570293
H	3.524551	-1.834910	-1.454306

RC: B + TeF_2-MA
E = -3183.14
H = -3057.72
G = -3102.16
N_{imag} = 0

C	2.382563	-1.272254	-0.604665
C	3.560000	-0.645120	-0.013009
C	3.886634	-0.622657	1.290273
C	1.349938	-1.823434	0.055573
H	4.782878	-0.110536	1.635145
H	3.292634	-1.136195	2.045585
H	1.311321	-1.866866	1.143020
H	0.500706	-2.247869	-0.476880
C	1.657012	1.428173	2.002246
C	0.578023	0.970385	1.352836
C	0.338692	1.198471	-0.072560
O	-0.672721	0.824056	-0.686862
O	1.307161	1.888966	-0.699868
C	1.112223	2.088191	-2.118243
Te	-2.562081	-0.721262	-0.339367
F	-4.009059	-1.988358	0.052875
F	-1.902538	-0.908902	1.485409
H	2.422770	2.010848	1.496640
H	1.785627	1.239103	3.066475
H	-0.202126	0.393802	1.845257
H	0.198309	2.663997	-2.301118
H	1.038890	1.120209	-2.626721
H	1.993406	2.639269	-2.453600
H	2.344818	-1.261054	-1.696803
H	4.224055	-0.136920	-0.716270

TS: B + TeF_2-MA
E = -3176.85
H = -3051.75
G = -3091.83
N_{imag} = 1, n = -325i cm^{-1}

C	2.439035	-1.182072	-0.586380
C	3.525766	-0.598715	0.097619
C	3.498966	-0.357272	1.464940
C	1.288666	-1.576854	0.045814
H	4.350429	0.134404	1.933689
H	2.951838	-1.035741	2.117102

H	1.257600	-1.774334	1.113198
H	0.407464	-1.885199	-0.515903
C	2.000556	1.093878	1.710800
C	0.799581	0.683590	1.135203
C	0.381186	1.118229	-0.175510
O	-0.745690	0.942789	-0.685051
O	1.332483	1.795362	-0.861886
C	0.969884	2.193843	-2.201207
Te	-2.558830	-0.630674	-0.311682
F	-3.952329	-1.955413	0.113780
F	-1.927200	-0.733467	1.532272
H	2.561940	1.890068	1.229831
H	2.078431	1.052923	2.796036
H	0.028552	0.181190	1.711785
H	0.105422	2.866620	-2.180750
H	0.726547	1.313880	-2.808069
H	1.850453	2.705412	-2.596752
H	2.460812	-1.170047	-1.677314
H	4.320918	-0.148988	-0.498856

P: B + TeF_2-MA
E = -3221.80
H = -3093.77
G = -3132.71
$\mathbf{N_{imag}}$ = 0

C	2.989134	-1.238739	-0.718341
C	3.533114	-0.095637	-0.289028
C	2.878847	0.795182	0.731493
C	1.647030	-1.748646	-0.265033
H	4.500285	0.220407	-0.684738
H	2.504145	1.712713	0.242715
H	3.628271	1.144452	1.457285
H	1.786754	-2.613993	0.403724
H	1.083089	-2.128839	-1.127685
C	1.742116	0.085269	1.469575
C	0.830981	-0.688797	0.488222
C	0.104001	0.296701	-0.398277
O	-0.657680	1.179971	0.011516
O	0.349339	0.161339	-1.706014
C	-0.324052	1.103010	-2.579704
Te	-1.597262	1.762350	2.276409
F	-2.306247	2.003485	4.087835
F	-1.437523	-0.154828	2.576443
H	2.155852	-0.651501	2.171383
H	1.151446	0.798154	2.058781
H	0.035046	-1.163405	1.079387
H	-0.020311	2.126165	-2.334025
H	-1.409588	1.009130	-2.469529
H	-0.005248	0.832067	-3.588152
H	3.527376	-1.851471	-1.443816

RC: B + PF_3-MA
E = -3398.31
H = -3269.72
G = -3315.88
$\mathbf{N_{imag}}$ = 0

C	-1.260281	-0.680757	1.361367
C	-2.469682	-0.489936	0.565408
C	-3.011164	-1.373488	-0.288202
C	-0.339403	-1.642483	1.191775
H	-1.090837	0.060960	2.145772
H	-3.908312	-1.130666	-0.854547
H	-2.593179	-2.369986	-0.430257
H	-0.429878	-2.396900	0.411359
H	0.542856	-1.696159	1.824727
C	-0.719389	-0.685528	-2.460158
C	0.430446	-0.626504	-1.779640
C	0.843817	0.515785	-0.943269
O	1.930755	0.603879	-0.385457
O	-0.100227	1.486215	-0.859513
C	0.256037	2.599338	-0.013290
H	-1.436472	0.131003	-2.431369
H	-0.965635	-1.554201	-3.068916
H	1.158663	-1.434933	-1.805693
H	1.148817	3.104969	-0.398714
H	0.456474	2.249804	1.006210
H	-0.608312	3.267379	-0.033679
P	3.927632	-1.212203	0.400537
F	2.938745	-2.302317	-0.254350
F	5.001582	-2.276815	0.966117
F	3.134636	-1.011821	1.781143
H	-2.965234	0.476085	0.688708

TS: B + PF_3-MA
E = -3390.75
H = -3262.48
G = -3303.62
N_{imag} = 1, n = -356i cm^{-1}

C	-1.391127	-0.657110	1.374146
C	-2.556275	-0.604051	0.582757
C	-2.723321	-1.403214	-0.537416
C	-0.351689	-1.509555	1.098580
H	-1.241429	0.131588	2.112810
H	-3.610555	-1.279192	-1.156924
H	-2.289699	-2.401244	-0.557144
H	-0.500530	-2.423490	0.530691
H	0.597674	-1.426900	1.622072
C	-1.170840	-0.758453	-1.860326
C	0.067831	-0.774270	-1.227203
C	0.702512	0.429931	-0.699661
O	1.883202	0.537687	-0.374187
O	-0.166657	1.479958	-0.610767
C	0.397733	2.680582	-0.047689
H	-1.639271	0.200261	-2.062668
H	-1.397054	-1.555909	-2.566127
H	0.740172	-1.620982	-1.330553
H	1.225033	3.045351	-0.667422
H	0.772227	2.489628	0.965174
H	-0.420341	3.405182	-0.028087
P	3.866601	-1.229391	0.314013
F	2.887329	-2.337711	-0.328126
F	4.980083	-2.280360	0.834350

F	3.108753	-1.078130	1.724629
H	-3.240463	0.231265	0.738837

P: B + PF_3-MA
E = -3439.80
H = -3308.63
G = -3348.64
N_{imag} = 0

C	1.995306	-1.619740	-0.060992
C	2.315930	-0.945900	-1.172871
C	2.761909	0.488718	-1.166145
C	2.004219	-1.019514	1.318000
H	2.253165	-1.448620	-2.139882
H	1.954966	1.127374	-1.563362
H	3.605850	0.616024	-1.860420
H	2.833206	-1.450608	1.902388
H	1.080008	-1.294219	1.845078
C	3.166589	0.953579	0.236786
C	2.137296	0.511275	1.292119
C	0.769684	1.168710	1.123695
O	-0.297727	0.653783	1.404283
O	0.876055	2.438826	0.662772
C	-0.382590	3.138062	0.511776
P	-1.120179	-1.086462	-0.951337
F	-0.646855	0.299378	-1.622336
F	-2.700213	-0.791334	-1.030374
F	-1.020018	-1.980249	-2.287600
H	3.300242	2.040450	0.260684
H	2.481618	0.853438	2.282323
H	-0.906156	3.191035	1.472879
H	-1.015121	2.619199	-0.216242
H	-0.117043	4.136145	0.155549
H	4.130647	0.496708	0.504302
H	1.707338	-2.670326	-0.135117

RC: B + AsF_3-MA
E = -3362.67
H = -3234.75
G = -3280.79
N_{imag} = 0

C	-1.280590	-0.674170	1.327686
C	-2.488395	-0.532913	0.520776
C	-2.962908	-1.417324	-0.372545
C	-0.345294	-1.630301	1.204919
H	-1.125063	0.101862	2.081072
H	-3.867544	-1.210621	-0.941237
H	-2.484377	-2.381434	-0.542658
H	-0.427615	-2.428803	0.468662
H	0.539678	-1.639766	1.836091
C	-0.697035	-0.701684	-2.425506
C	0.437361	-0.668566	-1.715620
C	0.865673	0.483549	-0.907871
O	1.958376	0.571359	-0.347113
O	-0.048482	1.475389	-0.848412
C	0.324644	2.601724	-0.024613
H	-1.381816	0.142326	-2.441732

H	-0.958027	-1.575828	-3.019679
H	1.136152	-1.502380	-1.696964
H	1.220866	3.089014	-0.424905
H	0.523884	2.268572	1.000242
H	-0.532517	3.278107	-0.056253
As	3.825683	-1.107401	0.408992
F	2.832759	-2.373847	-0.324221
F	5.041601	-2.244867	1.021772
F	2.943397	-1.022594	1.931110
H	-3.042308	0.398046	0.663791

TS: B + AsF_3-MA
E = -3356.00
H = -3228.39
G = -3269.76
N_{imag} = 1, n = -339i cm^{-1}

C	-1.392707	-0.647720	1.377025
C	-2.551950	-0.597201	0.575471
C	-2.715949	-1.403684	-0.540485
C	-0.353715	-1.504776	1.120528
H	-1.250850	0.142392	2.115889
H	-3.604573	-1.285888	-1.159200
H	-2.282011	-2.401793	-0.551037
H	-0.493423	-2.413556	0.542095
H	0.592596	-1.422033	1.649793
C	-1.182439	-0.757619	-1.865937
C	0.063700	-0.783487	-1.246705
C	0.698333	0.409074	-0.712758
O	1.888641	0.516089	-0.389792
O	-0.151005	1.466364	-0.613635
C	0.420842	2.656248	-0.032155
H	-1.645428	0.204593	-2.064978
H	-1.416444	-1.550786	-2.574020
H	0.726763	-1.638266	-1.341611
H	1.246694	3.027727	-0.649482
H	0.795979	2.445801	0.976271
H	-0.394966	3.382532	0.000748
As	3.730301	-1.120816	0.275851
F	2.714706	-2.404224	-0.399712
F	4.969823	-2.257678	0.855632
F	2.911659	-1.033620	1.838895
H	-3.237326	0.238748	0.723522

P: B + AsF_3-MA
E = -3404.79
H = -3274.31
G = -3314.69
N_{imag} = 0

C	1.898833	-1.527180	-0.066917
C	2.309335	-0.894143	-1.175168
C	2.862416	0.502041	-1.172079
C	1.909173	-0.905421	1.303609
H	2.244434	-1.405119	-2.137582
H	2.129705	1.179806	-1.640555
H	3.753939	0.544073	-1.815243
H	2.713194	-1.355933	1.907864

H	0.968753	-1.136845	1.822105
C	3.215028	0.981307	0.240344
C	2.107217	0.615460	1.247896
C	0.787951	1.319168	0.948974
O	-0.317646	0.801198	1.012988
O	0.972122	2.615052	0.629231
C	-0.240001	3.351242	0.320588
As	-1.012260	-0.668150	-1.254523
F	-0.313999	0.777982	-1.999710
F	-2.686080	-0.148094	-1.487828
F	-0.919125	-1.645092	-2.728278
H	3.397838	2.061215	0.247596
H	2.405699	0.980325	2.244117
H	-0.924767	3.327080	1.175167
H	-0.728187	2.908971	-0.553761
H	0.090658	4.370864	0.111252
H	4.139318	0.488115	0.574169
H	1.540044	-2.556095	-0.139573

RC: B + SbF_3-MA
E = -3359.71
H = -3231.98
G = -3278.11
$\mathbf{N_{imag}}$ = 0

C	-1.245911	-0.684049	1.315299
C	-2.465691	-0.545599	0.527548
C	-2.937897	-1.418917	-0.378750
C	-0.312555	-1.641568	1.182523
H	-1.077481	0.094785	2.063000
H	-3.853392	-1.215029	-0.930845
H	-2.448599	-2.372941	-0.572746
H	-0.409036	-2.447365	0.455972
H	0.583667	-1.644865	1.798018
C	-0.722882	-0.694306	-2.419102
C	0.415851	-0.666352	-1.714166
C	0.850527	0.489568	-0.920914
O	1.958023	0.590319	-0.380683
O	-0.058958	1.478644	-0.845097
C	0.323620	2.607511	-0.027172
H	-1.400227	0.155514	-2.440894
H	-0.991979	-1.569747	-3.007492
H	1.110036	-1.504114	-1.688909
H	1.205919	3.102630	-0.447914
H	0.548162	2.272870	0.991731
H	-0.540117	3.275835	-0.038951
Sb	3.871754	-1.035674	0.399240
F	2.761469	-2.390921	-0.433244
F	5.043653	-2.390884	1.159198
F	2.836094	-0.919378	2.027142
H	-3.031179	0.374399	0.694483

TS: B + SbF_3-MA
E = -3353.71
H = -3226.28
G = -3268.08
$\mathbf{N_{imag}}$ = 1, n = -325i cm^{-1}

C	-1.374098	-0.646789	1.376581
C	-2.535757	-0.621831	0.576291
C	-2.687874	-1.433503	-0.538105
C	-0.318441	-1.483558	1.126090
H	-1.250506	0.145441	2.116572
H	-3.585562	-1.339463	-1.147751
H	-2.229886	-2.420868	-0.549518
H	-0.432738	-2.389877	0.538261
H	0.625896	-1.378352	1.655978
C	-1.198742	-0.742110	-1.874805
C	0.060887	-0.764343	-1.282298
C	0.690427	0.421513	-0.742367
O	1.891940	0.535716	-0.437798
O	-0.152942	1.475416	-0.616153
C	0.420088	2.656570	-0.016192
H	-1.673874	0.218170	-2.054724
H	-1.436086	-1.527310	-2.590683
H	0.724860	-1.618124	-1.383181
H	1.233390	3.047686	-0.637801
H	0.810363	2.425063	0.981611
H	-0.400625	3.375340	0.044346
Sb	3.767505	-1.072425	0.235314
F	2.605301	-2.445745	-0.496215
F	4.954775	-2.430189	0.980471
F	2.820271	-0.915169	1.919388
H	-3.239683	0.198340	0.726362

P: B + SbF_3-MA
E = -3401.98
H = -3272.28
G = -3311.02
N_{imag} = 0

C	1.886760	-1.530571	-0.024849
C	2.271442	-0.913934	-1.153399
C	2.830011	0.478700	-1.184570
C	1.942076	-0.890991	1.336564
H	2.178245	-1.438082	-2.106405
H	2.087101	1.146932	-1.649973
H	3.706554	0.504547	-1.848638
H	2.768890	-1.331367	1.916843
H	1.022217	-1.119380	1.891961
C	3.215675	0.981635	0.210964
C	2.134823	0.629069	1.253250
C	0.808803	1.325772	0.972615
O	-0.292678	0.791893	1.036858
O	0.976410	2.622455	0.668703
C	-0.240876	3.349653	0.350892
Sb	-1.078219	-0.712764	-1.221064
F	-0.274277	0.901226	-1.942465
F	-2.857017	-0.120910	-1.728917
F	-0.755795	-1.702159	-2.861979
H	3.393377	2.062268	0.196146
H	2.458499	1.010562	2.235032
H	-0.942236	3.295574	1.190247
H	-0.697788	2.918599	-0.545594
H	0.080766	4.377909	0.173005

H	4.149953	0.498481	0.531056
H	1.528518	-2.561345	-0.073487
